# Associations of Serum Biomarkers of Fruit and Vegetable Intake With the Risk of Cause–Specific Mortality and All–Cause Mortality: A National Prospective Cohort Study

**DOI:** 10.3389/fnut.2022.874943

**Published:** 2022-05-11

**Authors:** Liyuan Pu, Ruijie Zhang, Xiaojie Wang, Tian Zhao, Hongpeng Sun, Liyuan Han

**Affiliations:** ^1^Key Laboratory of Diagnosis and Treatment of Digestive System Tumors of Zhejiang Province, Hwa Mei Hospital, University of Chinese Academy of Sciences, Ningbo, China; ^2^Department of Global Health, Ningbo Institute of Life and Health Industry, University of Chinese Academy of Sciences, Ningbo, China; ^3^Department of Neurology, Shenzhen Qianhai Shekou Free Trade Zone Hospital, Shenzhen, China; ^4^Department of Child and Adolescent Health and Social Medicine, School of Public Health, Medical College of Soochow University, Suzhou, China

**Keywords:** fruit, vegetables, biomarkers, vitamin C, cancer mortality, all–cause mortality

## Abstract

**Objective:**

The purpose of this study was to evaluate the associations of serum biomarkers of fruit and vegetable intake (vitamin C and carotenoids) with cause–specific mortality and all–cause mortality in a nationally representative sample of US adults.

**Methods:**

We analyzed data from 12,530 participants from the National Health and Nutrition Examination Survey III (1988–1994). The Cox proportional hazards models with restricted cubic spline were used for the analysis.

**Results:**

During 246,027 person–years of follow–up, 4,511 deaths occurred, including 1,395 deaths from cardiovascular disease, 1,072 deaths from heart disease, 323 deaths from cerebral disease, and 954 deaths from cancer. The serum vitamin C was significantly associated with the cancer and all–cause mortality, with hazard ratios (HRs) (95% CIs) for each one SD of 0.80 (0.71–0.91) and 0.91 (0.86–0.96). The serum alpha–carotene was significantly associated with the cancer mortality, with HRs (95% CIs) of 0.70 (0.54–0.90), 0.68 (0.48–0.95), 0.64 (0.43–0.95), and 0.44 (0.33–0.60) for comparisons of groups 2–5 with group 1 in model 2, respectively. The change for each one SD in the composite biomarker score, equivalent to a 0.483 times/month difference in total fruits and vegetables intake, gave an HR of 0.79 (0.69–0.90) for cancer mortality.

**Conclusion:**

Inverse associations were found between serum vitamin C, carotenoids, and composite biomarker score and outcomes expect for cerebral disease, heart disease, and cardiovascular disease mortality. This finding supports an increase in dietary fruit and vegetable intake as a primary prevention strategy for cancer and all–cause mortality.

## Introduction

Cardiovascular disease (CVD) and cancer are the major causes of deaths globally, causing an estimated 25.5 million deaths annually (1). Previously, evidence indicated that a higher intake of fruit and vegetable is associated with a reduced risk of CVD and cancer mortality (2, 3). However, fruit and vegetable intake has traditionally been assessed using dietary food frequency questionnaires, which are easily susceptible to measurement error and reporting biases (4). Thus, objective serum biomarkers of fruit and vegetable intake are indispensable to confirm these associations.

Vitamin C and carotenoids are considered as objective serum biomarkers of fruit and vegetable intake (4–6). Negative associations between the concentrations of these markers and CVD mortality or all–cause mortality risk in different populations were observed (7, 8). However, there is a lack of evidence for these associations in the general population of the United States (US), which has different lifestyles and dietary behaviors compared to other countries. Moreover, the associations between serum biomarkers of fruit and vegetable intake and cerebral disease, heart disease, CVD, and cancer mortality risk have not been previously evaluated.

Thus, we evaluated the associations between the baseline concentrations of serum biomarkers of fruit and vegetable intake (vitamin C and carotenoids) and cause–specific and all–cause mortality in the US nationally representative sample using data from the Third National Health and Nutrition Examination Survey (NHANES III) (1988–1994), which is a nationally representative cohort of the US population (9). We also constructed a composite biomarker score to systematically examine potential associations.

## Methods

### Study Population Sources

Participants in this study were recruited through the NHANES III (1988–1994), a nationally representative sample of noninstitutionalized civilians in the US (10). The design of the NHANES III used a multistage, stratified, clustered, and probability sampling method. It combines the results of home interviews and physical examinations and includes demographic, socioeconomic, dietary, health–related, and laboratory data. The survey was reviewed and approved by the National Health Statistics Research Ethics Review Board. Details of the survey design have been described elsewhere (10). All the participants of the survey signed an informed consent form. We conducted prospective analyses of adult participants (≥18 years). A flowchart of the participant inclusion process is shown in Supplementary Figure 1.

### Ascertainment of Outcomes

The survival and cause of death information of participants were obtained by a prospective follow–up survey that was implemented by the National Center for Health Statistics until 31 December 2015 (11). Mortality data were obtained from the National Death Index death certificate records, based on the participants' social security number, name, date of birth, etc. (12). The leading causes of death (UCOD_LEADING) are provided and based on the UCOD_113 variable, which was identified according to the International Classification of Diseases, Tenth revision (cancer: C00–C97; CVD: I00–I09, I11, I13, I20–I51, and I60–I69; heart disease: I00–I09, I11, I13, and I20–I51; and cerebral diseases: I60–I69) (13).

### Measurement of Serum Vitamin C and Carotenoid Concentrations

For the NHANES III, serum concentrations of vitamin C and carotenoids were measured using high–performance liquid chromatography with electrochemical detection (9). The lower limits of detection of alpha–carotene, beta–carotene, beta–cryptoxanthin, lycopene, and lutein/zeaxanthin were 0, 0.67, 0, 0.63, and 0.43 μg/dl, respectively (9).

### Demographic Characteristics

The demographic characteristics of the participants, including age (<40, 40– <60, and ≥ 60 years), sex (female, male), marital status (married/living as married, widowed/divorced, and separated/never married), body mass index (BMI) (<25, 25– <30, and ≥ 30 kg/m^2^), educational status (less than high school, high school, and more than high school), smoking status (current smoker, former smoker, and never smoker), alcohol consumption (0, 0– <6, 6– <12, 12– <24, and ≥ 24 times/week), physical activity (ideal, intermediate, and poor) (14), and income (<$20,000, $20,000–50,000, and > $50,000) were collected. Serum concentrations of high–density lipoprotein cholesterol (HDL–C) and low–density lipoprotein cholesterol (LDL–C) were calculated using the Friedewald equation (9). Total energy intake was calculated by summing the calories (kcal) from all the foods for every participant (15). Participants were classified into the two groups (low or high) according to HDL–C (<1.4 mmol/l and ≥ 1.4 mmol/l) and LDL–C (<3.7 mmol/l and ≥ 3.7 mmol/l) concentrations (16). Hypertension was defined as any self–reported history, medication history, a systolic blood pressure ≥ 130 mm Hg, or a diastolic blood pressure ≥ 80 mm Hg (17). Diabetes was defined as any self–reported history, medication history, or a serum glucose concentration ≥ 7.1 mmol/l (18).

### Statistical Analysis

Baseline information regarding serum biomarkers of fruit and vegetable intake was divided into quintiles (from the lowest to the highest concentration, group 1 to group 5). The sum of the five individual carotenoid concentrations was regarded as the serum total carotenoid concentration. The value was considered as composite biomarker score, which was generated by calculating the average of the standardized values of serum concentrations of vitamin C and the five individual carotenoids (19). We calculated the Spearman's correlations between serum concentrations of vitamin C, other vitamins (vitamin A, B12, and E), and carotenoids. Exposure factors, including serum vitamin C, carotenoid concentrations, and their composite biomarker score, were standardized. We used linear regression to evaluate the relationship and output standardized regression coefficients between serum vitamin C, carotenoids, and their composite biomarker scores and dietary covariates such as soft drink, fruit and vegetable juice, and red meat intake. Age, sex, physical activity, smoking status, marital status, educational status, alcohol consumption, total energy intake, HDL–C concentration, and BMI were adjusted in above analyses (16). We also evaluated the differences in total fruits and vegetable intake for each one SD higher composite biomarker score using linear regression.

Hazard ratios (HRs) and 95% CIs for all–cause and cause–specific mortality were determined by the Cox proportional hazards models. Serum biomarkers were divided into quintiles (group 1 to group 5, from the lowest to the highest concentration) and for each one SD change. Three models were used in this study. In model 1a, adjustments were made for age and sex. Model 1b included adjustments for age, sex, physical activity, smoking status, marital status, educational status (low, middle, and high), alcohol consumption (never, 0 to <6, 6– <12, 12– <24, and ≥ 24 times/week), total energy intake (continuous), and HDL–C concentration (continuous; only for carotenoids analyses). Model 2 included the same adjustments as model 1b, but was also adjusted for BMI (16). In model 2, we also used restricted cubic spline (four knots) to evaluate the relationship between serum vitamin C, carotenoids, and their composite biomarker scores and outcomes.

We excluded participants recruited within the first 2 years or the first 4 years or those with baseline cancer or CVD for sensitivity analyses. We further separately adjusted for hypertension, diabetes, and those with a history of cancer or CVD. The composite biomarker scores were also amended in this study, by excluding one biomarker at a time. Finally, we explored unmeasured confounding by calculating E–values (20). All the statistical analyses were performed using SAS version 9.4 statistical software (SAS Institute, Cary, North Carolina, USA). All the tests were two–sided and *P* < .05 was considered as statistically significant.

## Results

### Baseline Characteristics

Table 1 shows the baseline characteristics of serum biomarkers. In this study, the mean SD concentrations of serum vitamin C and total carotenoids in females were 40.41 (24.41) and 1.42 (0.65) μmol/l and in males were 34.17 (22.69) and 1.35 (0.61) μmol/l, respectively. The mean (SD) concentrations of serum vitamin C for the three age groups (<40, 40– <60, and ≥ 60 years) were 36.04 (23.06), 35.88 (23.44), and 42.47 (25.28) mmol/l, respectively, and those of serum total carotenoids for the three age groups were 1.31 (0.55), 1.44 (0.67), and 1.50 (0.75) μmol/l, respectively (Table 1). The serum concentrations of all the biomarkers of fruit and vegetable intake were all positively correlated with each other (Supplementary Table 1).

**Table 1:** Distribution of serum vitamin C and carotenoids by baseline characteristics.

**Subgroup**	**Serum vitamin C (mmol/L)**		**Total carotenoids (μmol/L)**		**Serum alpha carotene [μmol/L; Median(IQR^**a**^)]**	**Serum beta carotene [μmol/L;Median** **(IQR)]**	**Serum lycopene [μmol/L;Median** **(IQR)]**	**Serum lutein/zeaxanthin [μmol/L;Median** **(IQR)]**	**Serum beta cryptoxanthin [μmol/L;Median** **(IQR)]**	**Composite biomarker score [Median** **(IQR)]**
	**No**	**Mean(SD)**	**No**	**Mean(SD)**						
Sex										
Male	5846	34.17(22.69)	6093	1.35(0.61)	0.06(0.02,0.09)	0.20(0.13,0.35)	0.45(0.30,0.60)	0.33(0.25,0.46)	0.13(0.09,0.18)	−0.14(−0.44,0.24)
Female	6122	40.41(24.41)	6395	1.42(0.65)	0.07(0.04,0.11)	0.28(0.17,0.45)	0.39(0.28,0.54)	0.33(0.25,0.46)	0.13(0.09,0.20)	−0.04(−0.39,0.36)
Age (years)										
<40	5573	36.04(23.06)	5743	1.31(0.55)	0.06(0.02,0.09)	0.20(0.13,0.32)	0.45(0.32,0.60)	0.30(0.23,0.40)	0.13(0.09,0.18)	−0.15(−0.44,0.20)
<60	3040	35.88(23.44)	3150	1.44(0.67)	0.07(0.04,0.11)	0.26(0.17,0.43)	0.41(0.28,0.56)	0.35(0.26,0.49)	0.13(0.09,0.20)	−0.05(−0.41,0.37)
≥60	3355	42.47(25.28)	3595	1.50(0.75)	0.07(0.06,0.11)	0.34(0.20,0.54)	0.32(0.19,0.47)	0.39(0.28,0.54)	0.14(0.09,0.22)	0.03(−0.36,0.49)
BMI (kg/m^2^)										
<25	4786	38.81(24.02)	5008	1.43(0.66)	0.07(0.04,0.11)	0.28(0.17,0.43)	0.43(0.30,0.56)	0.33(0.25,0.46)	0.13(0.09,0.20)	−0.04(−0.38,0.38)
<30	4094	37.60(24.63)	4258	1.41(0.61)	0.07(0.04,0.09)	0.24(0.15,0.41)	0.41(0.28,0.60)	0.33(0.25,0.47)	0.13(0.09,0.20)	−0.06(−0.38,0.33)
≥30	3088	33.65(21.50)	3222	1.25(0.60)	0.06(0.02,0.07)	0.20(0.13,0.32)	0.39(0.26,0.56)	0.32(0.23,0.42)	0.11(0.07,0.16)	−0.21(−0.54,0.16)
Educational status										
low	5117	33.38(24.91)	5397	1.31(0.68)	0.06(0.02,0.09)	0.22(0.13,0.37)	0.35(0.24,0.50)	0.33(0.23,0.46)	0.11(0.07,0.18)	−0.22(−0.54,0.19)
Middle	5536	37.03(23.78)	5723	1.34(0.58)	0.06(0.02,0.09)	0.22(0.15,0.37)	0.43(0.30,0.58)	0.32(0.23,0.42)	0.13(0.09,0.18)	−0.10(−0.43,0.25)
High	1315	43.59(20.43)	1368	1.60(0.66)	0.09(0.06,0.15)	0.32(0.20,0.50)	0.45(0.34,0.60)	0.39(0.28,0.51)	0.16(0.11,0.24)	0.17(−0.18,0.56)
Smoking status										
Never	5732	43.09(22.81)	5991	1.50(0.68)	0.07(0.04,0.11)	0.28(0.17,0.47)	0.43(0.30,0.58)	0.35(0.26,0.47)	0.14(0.11,0.24)	0.05(−0.29,0.46)
Former	2819	39.98(22.50)	2955	1.45(0.65)	0.07(0.04,0.11)	0.30(0.17,0.45)	0.39(0.28,0.56)	0.35(0.26,0.49)	0.14(0.09,0.20)	0.00(−0.33,0.36)
Current	3417	27.18(22.73)	3542	1.17(0.48)	0.04(0.02,0.07)	0.19(0.11,0.28)	0.41(0.30,0.58)	0.28(0.21,0.39)	0.11(0.07,0.14)	−0.32(−0.60,0.00)
Alcohol consumption (times/week)										
0	9568	37.63(23.80)	10031	1.40(0.65)	0.07(0.04,0.11)	0.26(0.17,0.41)	0.41(0.28,0.56)	0.33(0.25,0.46)	0.13(0.09,0.20)	−0.08(−0.42,0.32)
0–<6	114	43.05(18.30)	119	1.45(0.51)	0.07(0.06,0.17)	0.28(0.15,0.48)	0.41(0.32,0.56)	0.39(0.28,0.49)	0.14(0.11,0.18)	0.05(−0.17,0.41)
6–<12	129	48.12(25.50)	130	1.63(0.69)	0.07(0.06,0.15)	0.32(0.24,0.54)	0.47(0.30,0.58)	0.42(0.32,0.62)	0.14(0.11,0.24)	0.09(−0.19,0.63)
12–<24	734	39.32(21.70)	744	1.43(0.56)	0.07(0.04,0.11)	0.24(0.15,0.39)	0.45(0.32,0.60)	0.35(0.28,0.49)	0.13(0.09,0.20)	0.01(−0.35,0.45)
≥24	1423	32.62(23.98)	1464	1.25(0.56)	0.06(0.02,0.07)	0.19(0.11,0.32)	0.43(0.30,0.56)	0.32(0.23,0.42)	0.11(0.09,0.16)	−0.22(−0.51,0.13)
Physical activity^b^										
Poor	2652	33.21(24.71)	2871	1.31(0.64)	0.06(0.02,0.09)	0.24(0.15,0.41)	0.34(0.22,0.48)	0.33(0.23,0.44)	0.13(0.07,0.18)	−0.20(−0.53,0.21)
Intermediate	5839	36.80(23.58)	6038	1.35(0.59)	0.06(0.02,0.09)	0.24(0.15,0.37)	0.43(0.30,0.58)	0.32(0.23,0.44)	0.13(0.09,0.18)	−0.10(−0.43,0.26)
Ideal	3477	40.12(23.26)	3579	1.47(0.69)	0.07(0.04,0.11)	0.28(0.17,0.45)	0.43(0.30,0.58)	0.35(0.25,0.47)	0.14(0.09,0.22)	0.00(−0.36,0.45)
Income ($)										
<20000	6518	34.6(23.79)	6895	1.30(0.66)	0.06(0.02,0.09)	0.22(0.13,0.37)	0.37(0.24,0.52)	0.32(0.23,0.44)	0.11(0.09,0.18)	−0.20(−0.53,0.21)
20000–50000	4035	38.1(24.35)	4153	1.40(0.62)	0.07(0.04,0.09)	0.26(0.15,0.41)	0.43(0.32,0.60)	0.33(0.25,0.44)	0.13(0.09,0.20)	−0.07(−0.38,0.30)
>50000	1415	40.58(21.90)	1440	1.50(0.58)	0.07(0.06,0.11)	0.28(0.17,0.43)	0.47(0.34,0.61)	0.35(0.28,0.49)	0.14(0.11,0.20)	0.06(−0.25,0.44)
Marry status										
Married/living as married	7077	37.12(23.58)	7365	1.40(0.61)	0.07(0.04,0.11)	0.26(0.15,0.41)	0.43(0.30,0.58)	0.33(0.25,0.46)	0.13(0.09,0.20)	−0.06(−0.39,0.32)
Widowed/divorced	1974	37.24(24.93)	2100	1.38(0.72)	0.07(0.02,0.11)	0.28(0.17,0.47)	0.34(0.22,0.50)	0.33(0.23,0.47)	0.13(0.09,0.20)	−0.14(−0.51,0.36)
Separated/never married	2917	37.61(23.53)	3023	1.32(0.63)	0.06(0.02,0.09)	0.20(0.13,0.34)	0.43(0.32,0.58)	0.30(0.23,0.42)	0.13(0.09,0.20)	−0.14(−0.45,0.21)
HDL–C										
Low (<1.4mmol/L)	7600	35.16(22.96)	7916	1.32(0.60)	0.06(0.02,0.09)	0.22(0.15,0.35)	0.41(0.28,0.58)	0.32(0.23,0.42)	0.13(0.09,0.18)	−0.15(−0.46,0.22)
High (≥1.4mmol/L)	4368	41.10(24.72)	4572	1.51(0.67)	0.07(0.04,0.11)	0.30(0.17,0.48)	0.43(0.30,0.56)	0.37(0.26,0.51)	0.14(0.11,0.22)	0.04(−0.33,0.49)
LDL–C										
Low (<3.7mmol/L)	10327	37.54(23.77)	10802	1.35(0.62)	0.06(0.02,0.09)	0.24(0.15,0.39)	0.41(0.28,0.56)	0.32(0.23,0.44)	0.13(0.09,0.20)	−0.12(−0.44,0.28)
High (≥3.7mmol/L)	1641	35.45(23.66)	1686	1.58(0.67)	0.07(0.04,0.11)	0.30(0.19,0.47)	0.48(0.32,0.65)	0.37(0.28,0.51)	0.14(0.09,0.22)	0.06(−0.31,0.48)
Hypertension										
Yes	3316	37.94(25.37)	3504	1.41(0.73)	0.07(0.04,0.09)	0.28(0.15,0.45)	0.35(0.22,0.52)	0.37(0.26,0.51)	0.13(0.09,0.20)	−0.10(−0.45,0.38)
No	8652	37.06(23.31)	8984	1.37(0.60)	0.06(0.02,0.09)	0.24(0.15,0.39)	0.43(0.30,0.58)	0.32(0.25,0.44)	0.13(0.09,0.20)	−0.09(−0.41,0.29)
Diabetes										
No	10891	37.36(23.95)	11347	1.38(0.62)	0.06(0.02,0.09)	0.24(0.15,0.39)	0.41(0.30,0.58)	0.33(0.25,0.46)	0.13(0.09,0.20)	−0.09(−0.42,0.31)
Yes	1077	35.61(20.67)	1141	1.39(0.75)	0.07(0.04,0.09)	0.26(0.17,0.43)	0.35(0.22,0.48)	0.35(0.25,0.49)	0.13(0.09,0.20)	−0.10(−0.45,0.26)

a *The full name of IQR is interquartile range*.

b *Physical activity included walking, jogging or running, bicycling, swimming, aerobics or aerobic dancing, other dancing, calisthenics, gardening or yard work, and other sports. Ideal was regard as engaging in physical activities with 3 ≤ METS < 6 and ≥ 5 times / week or physical activities with METS ≥ 6 and 3.0 times / week. The difference between ideal and no physical activity was taken as intermediate. No physical activity was taken as poor*.

### Associations of Serum Concentrations of Biomarkers and Demographic Characteristics

After multivariate adjustment, educational status was positively associated with serum vitamin C, total carotenoid concentration, and composite biomarker score. Meanwhile, the inverse associations were found between BMI and current smoking status (compared with never smoking) and the serum concentrations of vitamin C, total carotenoid concentration, and composite biomarker score (Supplementary Table 2).

**Figure 1:**
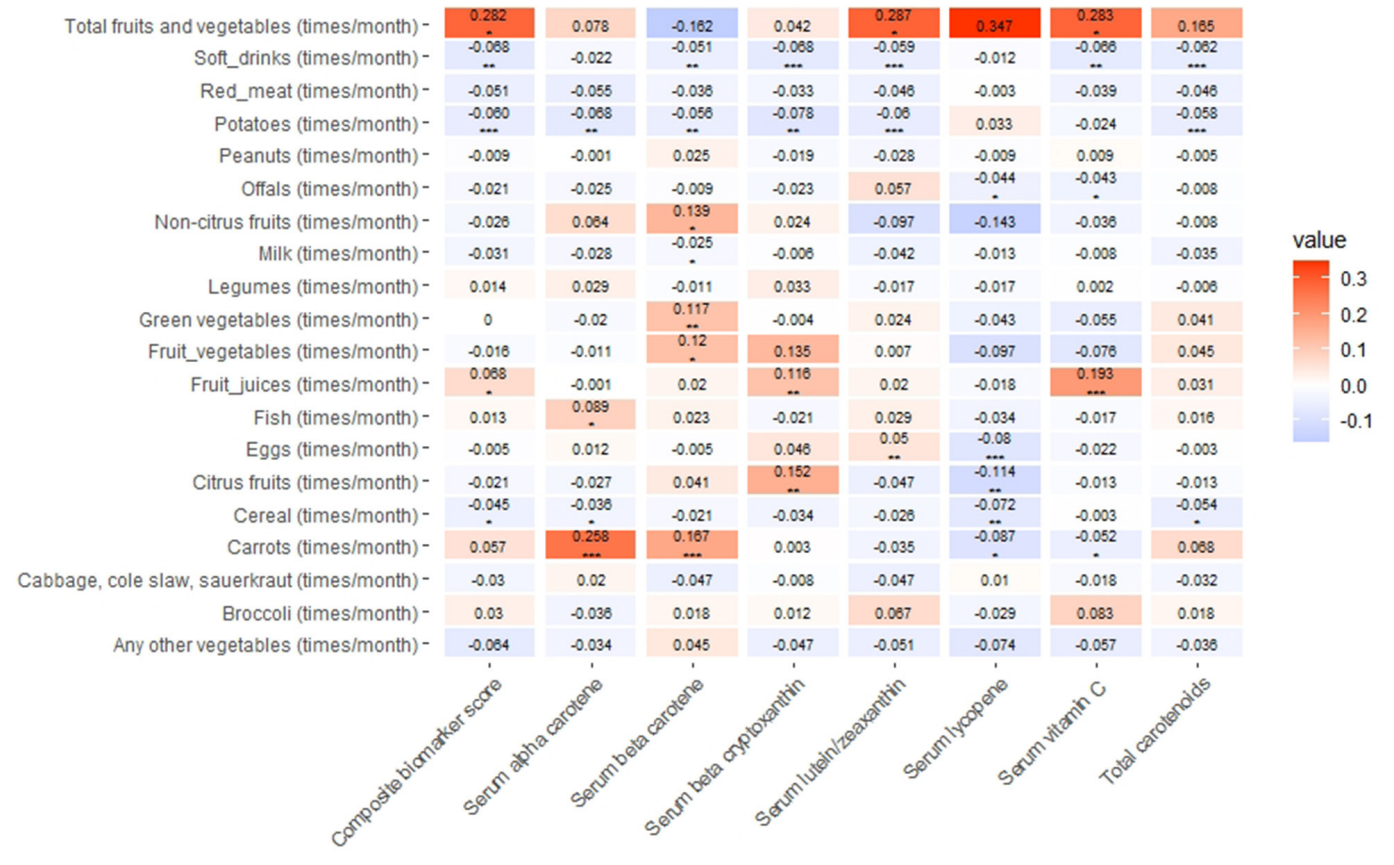
Association of dietary factors with serum vitamin C, carotenoids, and their composite biomarker scores in the Third National Health and Nutrition Examination Survey (NHANES III). Linear regression was used to obtain the estimate values of an association between dietary factors and vitamin C, carotenoids, or the composite biomarker score (in SD units), adjusting for demographic and lifestyle factors. The positive associations and negative associations of values were expressed in a red scale and blue scale, respectively. *P < 0.05; ^**^P < 0.01; ^***^P < 0.001. Each one time/month higher intake of total fruits and vegetables was associated with 0.282 higher level of the composite biomarker score; conversely, every one SD higher composite biomarker score was associated with a 0.483 times/months higher intake of total fruits and vegetables. Each one time/month higher intake of fruits and vegetable was associated with 0.283 higher level of serum vitamin C; conversely, every one SD serum vitamin C was associated with a 0.435 times/months higher intake of fruits and vegetables in this figure.

The positive association was found between monthly intake of fruit juices and serum vitamin C, beta–cryptoxanthin, and the composite biomarker score (*P* < 0.05, Figure 1). The number of times per month that soft drinks were consumed was negatively associated with serum vitamin C, total carotenoids, and the composite biomarker score (*P* < 0.05, Figure 1). Each one time/month higher intake of total fruits and vegetables was associated with an increase of 0.282 in the composite biomarker score. Conversely, each one (SD) increase in the composite biomarker score was associated with a 0.483 times/month increase in the intake of total fruits and vegetables (Figure 1 and Supplementary Table 2). Each one time/month increase in the intake of total fruits and vegetables was associated with a 0.283 μmol/l increase in serum vitamin C concentration. Conversely, each one SD increase in serum vitamin C concentration was associated with a 0.435 times/month increase in the intake of total fruits and vegetables (Figure 1 and Supplementary Table 2).

### Associations of Serum Biomarkers of Fruit and Vegetable Intake With Outcomes

Table 2 shows the HRs and 95% CIs for the associations between serum concentrations of biomarkers of fruit and vegetable intake and outcomes. The medians for total fruit and vegetable intake were 39, 50, 57, 68, and 89 times/month for lowest concentrations (group 1), 2, 3, 4, and 5 (highest concentrations) of the composite biomarker score, respectively (Table 2). Higher serum concentrations of vitamin C were associated with lower cancer mortality. The HRs (95% CIs) for each one SD increase in serum vitamin C concentration were 0.70 (0.62–0.78), 0.80 (0.71–0.91), and 0.80 (0.71–0.91) in models 1a, 1b, and 2, respectively. The serum vitamin C concentration was significantly associated with the cancer mortality, with HRs (95% CIs) of 0.68 (0.49–0.93), 0.51 (0.35–0.75), and 0.53 (0.38–0.75) for comparisons of groups 3, 4, and 5, respectively, with group 1 in model 2 (*P* < 0.001 for the trend; Table 2).

**Table 2:** Prospective associations between serum biomarkers levels of fruit and vegetable intake and outcomes.

**All–cause and cause–specific mortality**	**Biomarkers**	**Hazard ratio (95% confidence interval)**						
		**Group 1**	**Group 2**	**Group 3**	**Group 4**	**Group 5**	**For each one standard deviation**	***P*–value for trend**
CVD Mortality	Serum vitamin C (median, mmol/L)	10.83	26.20	37.08	47.93	55.21		
	Model1a	1	0.62 (0.50–0.77)	0.60 (0.47–0.76)	0.48 (0.37–0.61)	0.49 (0.39–0.63)	0.84 (0.77–0.92)	<0.001
	Model1b	1	0.69 (0.55–0.85)	0.72 (0.57–0.92)	0.62 (0.47–0.80)	0.64 (0.50–0.82)	0.92 (0.84–1.01)	0.002
	Model2	1	0.68 (0.55–0.85)	0.72 (0.57–0.92)	0.62 (0.47–0.80)	0.65 (0.50–0.83)	0.93 (0.85–1.01)	0.003
	Total carotenoids (median, μmol/L)	0.75	1.04	1.28	1.55	2.13		
	Model1a	1	0.78 (0.59–1.04)	0.77 (0.58–1.03)	0.82 (0.65–1.04)	0.75 (0.59–0.95)	0.92 (0.83–1.01)	0.055
	Model1b	1	0.89 (0.68–1.16)	0.90 (0.69–1.19)	1.01 (0.80–1.27)	0.98 (0.78–1.23)	0.99 (0.90–1.09)	0.763
	Model2	1	0.89 (0.69–1.16)	0.92 (0.70–1.19)	1.02 (0.82–1.29)	1.00 (0.79–1.26)	1.00 (0.91–1.10)	0.623
	Serum alpha carotene (median, μmol/L)	0.01	0.04	0.06	0.07	0.13		
	Model1a	1	0.76 (0.59–0.98)	0.77 (0.59–1.01)	0.67 (0.51–0.90)	0.62 (0.46–0.82)	0.82 (0.72–0.92)	0.003
	Model1b	1	0.83 (0.65–1.05)	0.89 (0.68–1.17)	0.82 (0.62–1.08)	0.79 (0.58–1.08)	0.90 (0.80–1.01)	0.218
	Model2	1	0.83 (0.65–1.05)	0.90 (0.69–1.17)	0.83 (0.63–1.09)	0.81 (0.59–1.10)	0.91 (0.81–1.02)	0.275
	Serum beta carotene (median, μmol/L)	0.11	0.17	0.23	0.32	0.54		
	Model1a	1	0.75 (0.56–1.00)	0.66 (0.49–0.88)	0.66 (0.52–0.83)	0.62 (0.47–0.82)	0.89 (0.82–0.97)	0.003
	Model1b	1	0.79 (0.58–1.07)	0.73 (0.55–0.96)	0.79 (0.62–1.00)	0.77 (0.57–1.03)	0.95 (0.89–1.02)	0.175
	Model2	1	0.79 (0.58–1.07)	0.74 (0.56–0.97)	0.79 (0.63–1.01)	0.79 (0.58–1.06)	0.96 (0.89–1.03)	0.242
	Serum lycopene (median, μmol/L)	0.27	0.36	0.43	0.48	0.53		
	Model1a	1	0.99 (0.80–1.22)	0.77 (0.59–0.99)	0.84 (0.64–1.10)	0.88 (0.70–1.11)	0.99 (0.89–1.10)	0.076
	Model1b	1	1.06 (0.86–1.31)	0.85 (0.67–1.09)	0.97 (0.74–1.26)	1.04 (0.84–1.29)	1.05 (0.95–1.16)	0.854
	Model2	1	1.06 (0.86–1.31)	0.85 (0.66–1.09)	0.97 (0.75–1.27)	1.04 (0.84–1.29)	1.05 (0.95–1.16)	0.856
	Serum lutein/zeaxanthin (median, μmol/L)	0.20	0.27	0.32	0.39	0.54		
	Model1a	1	0.78 (0.63–0.96)	0.90 (0.71–1.15)	0.79 (0.59–1.04)	0.90 (0.70–1.16)	1.00 (0.90–1.11)	0.648
	Model1b	1	0.79 (0.62–1.01)	1.02 (0.80–1.31)	0.89 (0.67–1.18)	1.05 (0.80–1.36)	1.04 (0.95–1.14)	0.372
	Model2	1	0.80 (0.63–1.01)	1.04 (0.81–1.33)	0.90 (0.68–1.20)	1.06 (0.82–1.38)	1.04 (0.95–1.15)	0.302
	Serum beta cryptoxanthin (median, μmol/L)	0.06	0.09	0.12	0.15	0.24		
	Model1a	1	0.94 (0.72–1.24)	0.87 (0.65–1.16)	0.80 (0.60–1.05)	0.68 (0.54–0.86)	0.88 (0.80–0.97)	0.006
	Model1b	1	1.07 (0.81–1.40)	1.02 (0.76–1.36)	0.97 (0.73–1.28)	0.89 (0.69–1.14)	0.97 (0.89–1.06)	0.444
	Model2	1	1.06 (0.81–1.39)	1.02 (0.76–1.37)	0.97 (0.74–1.27)	0.90 (0.70–1.15)	0.97 (0.89–1.06)	0.469
	Composite biomarker score (median)	−0.67	−0.36	−0.09	0.22	0.75		
	Model1a	1	0.82 (0.61–1.10)	0.78 (0.63–0.97)	0.71 (0.51–0.98)	0.61 (0.47–0.79)	0.86 (0.78–0.95)	0.001
	Model1b	1	0.93 (0.69–1.25)	0.94 (0.74–1.18)	0.89 (0.65–1.22)	0.83 (0.64–1.08)	0.96 (0.87–1.05)	0.172
	Model2	1	0.93 (0.69–1.25)	0.94 (0.75–1.18)	0.90 (0.66–1.22)	0.84 (0.65–1.10)	0.96 (0.87–1.06)	0.220
Heart Mortality	Serum vitamin C (median, mmol/L)	10.83	26.20	37.08	47.93	55.21		
	Model1a	1	0.56 (0.44–0.72)	0.57 (0.43–0.76)	0.43 (0.32–0.58)	0.46 (0.35–0.61)	0.82 (0.74–0.92)	<0.001
	Model1b	1	0.62 (0.49–0.79)	0.71 (0.54–0.94)	0.58 (0.43–0.77)	0.62 (0.46–0.84)	0.92 (0.82–1.03)	0.005
	Model2	1	0.62 (0.49–0.79)	0.71 (0.54–0.94)	0.57 (0.43–0.77)	0.63 (0.47–0.85)	0.92 (0.83–1.03)	0.006
	Total carotenoids (median, μmol/L)	0.75	1.04	1.28	1.55	2.13		
	Model1a	1	0.80 (0.59–1.07)	0.70 (0.52–0.95)	0.84 (0.62–1.12)	0.72 (0.54–0.95)	0.92 (0.81–1.04)	0.078
	Model1b	1	0.93 (0.71–1.22)	0.84 (0.63–1.14)	1.07 (0.80–1.44)	1.00 (0.76–1.31)	1.01 (0.90–1.13)	0.680
	Model2	1	0.94 (0.72–1.23)	0.86 (0.64–1.15)	1.09 (0.82–1.46)	1.02 (0.78–1.34)	1.02 (0.91–1.14)	0.566
	Serum alpha carotene (median, μmol/L)	0.01	0.04	0.06	0.07	0.13		
	Model1a	1	0.76 (0.57–1.01)	0.74 (0.52–1.06)	0.62 (0.43–0.89)	0.61 (0.43–0.86)	0.80 (0.69–0.93)	0.007
	Model1b	1	0.84 (0.64–1.10)	0.89 (0.63–1.25)	0.78 (0.55–1.10)	0.83 (0.57–1.22)	0.90 (0.79–1.04)	0.334
	Model2	1	0.84 (0.64–1.10)	0.89 (0.63–1.25)	0.79 (0.56–1.12)	0.85 (0.58–1.24)	0.91 (0.79–1.05)	0.399
	Serum beta carotene (median, μmol/L)	0.11	0.17	0.23	0.32	0.54		
	Model1a	1	0.80 (0.56–1.15)	0.75 (0.52–1.08)	0.66 (0.49–0.88)	0.63 (0.45–0.89)	0.89 (0.80–0.99)	0.005
	Model1b	1	0.86 (0.61–1.22)	0.85 (0.60–1.20)	0.82 (0.62–1.10)	0.83 (0.59–1.16)	0.96 (0.88–1.06)	0.309
	Model2	1	0.86 (0.61–1.22)	0.86 (0.61–1.21)	0.83 (0.62–1.11)	0.85 (0.61–1.20)	0.97 (0.88–1.07)	0.402
	Serum lycopene (median, μmol/L)	0.27	0.36	0.43	0.48	0.53		
	Model1a	1	1.01 (0.77–1.33)	0.78 (0.59–1.04)	0.83 (0.61–1.12)	0.95 (0.73–1.25)	1.01 (0.89–1.16)	0.276
	Model1b	1	1.11 (0.86–1.45)	0.90 (0.69–1.18)	0.99 (0.73–1.34)	1.18 (0.91–1.53)	1.09 (0.96–1.24)	0.547
	Model2	1	1.11 (0.86–1.45)	0.90 (0.68–1.18)	1.00 (0.74–1.34)	1.17 (0.90–1.52)	1.09 (0.96–1.24)	0.545
	Serum lutein/zeaxanthin (median, μmol/L)	0.20	0.27	0.32	0.39	0.54		
	Model1a	1	0.74 (0.60–0.93)	0.86 (0.66–1.12)	0.78 (0.61–1.00)	0.85 (0.62–1.18)	1.00 (0.89–1.13)	0.565
	Model1b	1	0.76 (0.61–0.95)	1.01 (0.77–1.32)	0.92 (0.71–1.19)	1.05 (0.76–1.44)	1.06 (0.95–1.18)	0.344
	Model2	1	0.77 (0.61–0.96)	1.02 (0.79–1.34)	0.93 (0.72–1.21)	1.07 (0.78–1.46)	1.06 (0.95–1.18)	0.281
	Serum beta cryptoxanthin (median, μmol/L)	0.06	0.09	0.12	0.15	0.24		
	Model1a	1	0.97 (0.72–1.29)	0.98 (0.72–1.35)	0.76 (0.57–1.02)	0.62 (0.46–0.83)	0.84 (0.73–0.95)	0.002
	Model1b	1	1.12 (0.85–1.48)	1.19 (0.87–1.63)	0.97 (0.72–1.29)	0.85 (0.64–1.14)	0.94 (0.84–1.06)	0.380
	Model2	1	1.11 (0.84–1.47)	1.19 (0.87–1.64)	0.97 (0.73–1.29)	0.86 (0.64–1.15)	0.95 (0.85–1.06)	0.405
	Composite biomarker score (median)	−0.67	−0.36	−0.09	0.22	0.75		
	Model1a	1	0.82 (0.58–1.15)	0.79 (0.59–1.06)	0.71 (0.50–1.01)	0.59 (0.43–0.80)	0.86 (0.76–0.97)	0.002
	Model1b	1	0.93 (0.66–1.33)	0.98 (0.72–1.33)	0.93 (0.66–1.32)	0.85 (0.63–1.16)	0.97 (0.87–1.09)	0.355
	Model2	1	0.93 (0.66–1.33)	0.99 (0.73–1.34)	0.94 (0.67–1.32)	0.87 (0.64–1.18)	0.98 (0.88–1.10)	0.428
Cerebral Mortality	Serum vitamin C (median, mmol/L)	10.83	26.20	37.08	47.93	55.21		
	Model1a	1	0.93 (0.44–1.96)	0.73 (0.41–1.32)	0.73 (0.37–1.46)	0.67 (0.39–1.15)	0.90 (0.76–1.08)	0.100
	Model1b	1	1.04 (0.49–2.20)	0.81 (0.46–1.42)	0.84 (0.39–1.78)	0.76 (0.42–1.38)	0.93 (0.78–1.12)	0.265
	Model2	1	1.03 (0.49–2.18)	0.81 (0.46–1.42)	0.84 (0.39–1.78)	0.76 (0.42–1.38)	0.94 (0.78–1.12)	0.259
	Total carotenoids (median, μmol/L)	0.75	1.04	1.28	1.55	2.13		
	Model1a	1	0.71 (0.35–1.46)	1.06 (0.54–2.07)	0.77 (0.42–1.42)	0.89 (0.42–1.90)	0.92 (0.78–1.08)	0.817
	Model1b	1	0.74 (0.36–1.51)	1.13 (0.58–2.21)	0.80 (0.44–1.47)	0.92 (0.43–1.95)	0.92 (0.79–1.07)	0.873
	Model2	1	0.74 (0.37–1.50)	1.14 (0.59–2.19)	0.81 (0.45–1.47)	0.93 (0.45–1.92)	0.92 (0.79–1.07)	0.906
	Serum alpha carotene (median, μmol/L)	0.01	0.04	0.06	0.07	0.13		
	Model1a	1	0.79 (0.42–1.48)	0.89 (0.52–1.54)	0.91 (0.49–1.67)	0.65 (0.3–1.39)	0.87 (0.66–1.14)	0.389
	Model1b	1	0.8 (0.41–1.56)	0.94 (0.54–1.64)	0.97 (0.52–1.80)	0.67 (0.3–1.49)	0.87 (0.67–1.14)	0.482
	Model2	1	0.8 (0.41–1.55)	0.95 (0.55–1.63)	0.97 (0.53–1.79)	0.68 (0.31–1.47)	0.88 (0.68–1.14)	0.488
	Serum beta carotene (median, μmol/L)	0.11	0.17	0.23	0.32	0.54		
	Model1a	1	0.57 (0.24–1.36)	0.37 (0.19–0.72)	0.64 (0.34–1.21)	0.56 (0.26–1.18)	0.91 (0.78–1.06)	0.329
	Model1b	1	0.56 (0.23–1.37)	0.38 (0.19–0.75)	0.65 (0.34–1.24)	0.57 (0.27–1.21)	0.92 (0.79–1.07)	0.386
	Model2	1	0.56 (0.23–1.37)	0.38 (0.19–0.75)	0.66 (0.35–1.23)	0.58 (0.28–1.19)	0.92 (0.80–1.06)	0.387
	Serum lycopene (median, μmol/L)	0.27	0.36	0.43	0.48	0.53		
	Model1a	1	0.89 (0.54–1.48)	0.70 (0.44–1.12)	0.88 (0.50–1.57)	0.64 (0.33–1.24)	0.89 (0.71–1.12)	0.208
	Model1b	1	0.91 (0.55–1.49)	0.71 (0.44–1.14)	0.89 (0.51–1.57)	0.64 (0.34–1.20)	0.89 (0.72–1.11)	0.200
	Model2	1	0.9 (0.55–1.48)	0.71 (0.44–1.14)	0.90 (0.51–1.57)	0.64 (0.34–1.20)	0.89 (0.72–1.11)	0.199
	Serum lutein/zeaxanthin (median, μmol/L)	0.20	0.27	0.32	0.39	0.54		
	Model1a	1	0.93 (0.40–2.17)	1.09 (0.56–2.12)	0.81 (0.35–1.85)	1.10 (0.60–2.03)	0.99 (0.89–1.10)	0.835
	Model1b	1	0.93 (0.40–2.18)	1.06 (0.54–2.10)	0.79 (0.35–1.80)	1.04 (0.55–1.97)	0.97 (0.87–1.08)	0.998
	Model2	1	0.93 (0.40–2.20)	1.07 (0.54–2.13)	0.80 (0.35–1.84)	1.05 (0.55–2.01)	0.97 (0.87–1.09)	0.972
	Serum beta cryptoxanthin (median, μmol/L)	0.06	0.09	0.12	0.15	0.24		
	Model1a	1	0.85 (0.44–1.64)	0.46 (0.24–0.90)	0.93 (0.55–1.57)	0.9 (0.54–1.50)	1.02 (0.88–1.18)	0.712
	Model1b	1	0.89 (0.47–1.69)	0.49 (0.25–0.94)	0.99 (0.58–1.68)	0.96 (0.58–1.60)	1.05 (0.91–1.20)	0.914
	Model2	1	0.89 (0.47–1.69)	0.49 (0.26–0.93)	0.99 (0.58–1.67)	0.97 (0.58–1.60)	1.05 (0.92–1.20)	0.930
	Composite biomarker score(median)	−0.67	−0.36	−0.09	0.22	0.75		
	Model1a	1	0.83 (0.42–1.65)	0.74 (0.34–1.57)	0.69 (0.36–1.32)	0.71 (0.33–1.52)	0.88 (0.74–1.06)	0.289
	Model1b	1	0.89 (0.45–1.76)	0.78 (0.37–1.67)	0.76 (0.39–1.45)	0.75 (0.34–1.63)	0.89 (0.75–1.07)	0.349
	Model2	1	0.89 (0.45–1.76)	0.79 (0.37–1.66)	0.76 (0.4–1.44)	0.75 (0.35–1.60)	0.9 (0.76–1.06)	0.343
Cancer Mortality	Serum vitamin C (median, mmol/L)	10.83	26.20	37.08	47.93	55.21		
	Model1a	1	0.73 (0.55–0.96)	0.52 (0.38–0.70)	0.36 (0.26–0.50)	0.38 (0.28–0.50)	0.70 (0.62–0.78)	<0.001
	Model1b	1	0.85 (0.64–1.12)	0.68 (0.49–0.93)	0.51 (0.35–0.75)	0.54 (0.39–0.74)	0.80 (0.71–0.91)	<0.001
	Model2	1	0.85 (0.64–1.12)	0.68 (0.49–0.93)	0.51 (0.35–0.75)	0.53 (0.38–0.75)	0.80 (0.71–0.91)	<0.001
	Total carotenoids (median, μmol/L)	0.75	1.04	1.28	1.55	2.13		
	Model1a	1	0.77 (0.52–1.12)	0.70 (0.51–0.97)	0.52 (0.39–0.70)	0.43 (0.33–0.57)	0.76 (0.66–0.89)	<0.001
	Model1b	1	0.87 (0.59–1.28)	0.85 (0.60–1.19)	0.65 (0.48–0.89)	0.60 (0.45–0.80)	0.87 (0.75–1.00)	<0.001
	Model2	1	0.86 (0.58–1.28)	0.84 (0.60–1.19)	0.65 (0.47–0.89)	0.60(0.44–0.81)	0.86 (0.74–1.00)	<0.001
	Serum alpha carotene (median, μmol/L)	0.01	0.04	0.06	0.07	0.13		
	Model1a	1	0.64 (0.49–0.84)	0.57 (0.42–0.78)	0.51 (0.35–0.75)	0.32 (0.25–0.42)	0.65 (0.55–0.76)	<0.001
	Model1b	1	0.70 (0.54–0.90)	0.68 (0.48–0.95)	0.64 (0.44–0.95)	0.45 (0.34–0.60)	0.77 (0.67–0.89)	<0.001
	Model2	1	0.70 (0.54–0.90)	0.68 (0.48–0.95)	0.64 (0.43–0.95)	0.44 (0.33–0.60)	0.77 (0.66–0.89)	<0.001
	Serum beta carotene (median, μmol/L)	0.11	0.17	0.23	0.32	0.54		
	Model1a	1	0.78 (0.56–1.08)	0.64 (0.47–0.89)	0.60 (0.46–0.79)	0.42 (0.32–0.54)	0.81 (0.68–0.97)	<0.001
	Model1b	1	0.82 (0.59–1.14)	0.73 (0.53–1.00)	0.73 (0.55–0.97)	0.57 (0.43–0.75)	0.92 (0.79–1.07)	<0.001
	Model2	1	0.82 (0.59–1.13)	0.72 (0.52–1.00)	0.73 (0.55–0.96)	0.56 (0.42–0.74)	0.92 (0.79–1.07)	<0.001
	Serum lycopene (median, μmol/L)	0.27	0.36	0.43	0.48	0.53		
	Model1a	1	0.92 (0.68–1.23)	0.62 (0.46–0.82)	0.62 (0.46–0.84)	0.72 (0.55–0.94)	0.85 (0.78–0.93)	0.001
	Model1b	1	0.96 (0.71–1.29)	0.67 (0.50–0.89)	0.68 (0.50–0.92)	0.82 (0.61–1.09)	0.89 (0.81–0.98)	0.024
	Model2	1	0.96 (0.71–1.29)	0.67 (0.50–0.89)	0.68 (0.50–0.93)	0.82 (0.61–1.09)	0.89 (0.81–0.98)	0.024
	Serum lutein/zeaxanthin (median, mmol/L)	0.20	0.27	0.32	0.39	0.54		
	Model1a	1	0.83 (0.59–1.18)	0.61 (0.43–0.86)	0.66 (0.46–0.93)	0.55 (0.42–0.73)	0.90 (0.74–1.10)	<0.001
	Model1b	1	0.87 (0.61–1.24)	0.70 (0.49–0.99)	0.77 (0.54–1.11)	0.69 (0.52–0.90)	0.98 (0.82–1.17)	0.007
	Model2	1	0.87 (0.61–1.24)	0.70 (0.49–0.99)	0.77 (0.53–1.12)	0.68 (0.52–0.90)	0.98 (0.82–1.17)	0.008
	Serum beta cryptoxanthin (median, mmol/L)	0.06	0.09	0.12	0.15	0.24		
	Model1a	1	0.77 (0.62–0.97)	0.60 (0.44–0.81)	0.57 (0.43–0.75)	0.39 (0.29–0.52)	0.63 (0.54–0.73)	<0.001
	Model1b	1	0.91 (0.72–1.15)	0.73 (0.54–0.99)	0.75 (0.57–0.99)	0.57 (0.42–0.78)	0.76 (0.65–0.89)	<0.001
	Model2	1	0.91 (0.72–1.15)	0.73 (0.54–0.99)	0.75 (0.57–0.99)	0.57 (0.42–0.78)	0.76 (0.65–0.89)	<0.001
	Composite biomarker score (median)	−0.67	−0.36	−0.09	0.22	0.75		
	Model1a	1	0.63 (0.46–0.88)	0.48 (0.36–0.63)	0.41 (0.30–0.55)	0.31 (0.24–0.39)	0.69 (0.61–0.78)	<0.001
	Model1b	1	0.74 (0.53–1.02)	0.59 (0.44–0.78)	0.53 (0.38–0.74)	0.44 (0.34–0.57)	0.79 (0.70–0.90)	<0.001
	Model2	1	0.74 (0.53–1.02)	0.58 (0.44–0.77)	0.53 (0.38–0.74)	0.44 (0.33–0.57)	0.79 (0.69–0.90)	<0.001
All–cause mortality	Serum vitamin C (median, mmol/L)	10.83	26.20	37.08	47.93	55.21		
	Model1a	1	0.77 (0.68–0.88)	0.64 (0.56–0.74)	0.55 (0.48–0.63)	0.52 (0.46–0.60)	0.82 (0.77–0.86)	<0.001
	Model1b	1	0.86 (0.75–0.99)	0.80 (0.69–0.93)	0.73 (0.62–0.86)	0.69 (0.60–0.80)	0.90 (0.85–0.95)	<0.001
	Model2	1	0.86 (0.74–0.99)	0.80 (0.69–0.93)	0.73 (0.62–0.86)	0.70 (0.60–0.81)	0.91 (0.86–0.96)	<0.001
	Total carotenoids (median, μmol/L)	0.75	1.04	1.28	1.55	2.13		
	Model1a	1	0.75 (0.66–0.84)	0.67 (0.57–0.78)	0.61 (0.53–0.69)	0.51 (0.44–0.60)	0.81 (0.76–0.86)	<0.001
	Model1b	1	0.83 (0.74–0.94)	0.77 (0.66–0.90)	0.73 (0.63–0.85)	0.66 (0.57–0.76)	0.88 (0.83–0.94)	<0.001
	Model2	1	0.84 (0.75–0.94)	0.78 (0.67–0.90)	0.74 (0.63–0.86)	0.67 (0.58–0.77)	0.89 (0.84–0.94)	<0.001
	Serum alpha carotene (median, μmol/L)	0.01	0.04	0.06	0.07	0.13		
	Model1a	1	0.68 (0.57–0.81)	0.58 (0.49–0.69)	0.54 (0.45–0.64)	0.43 (0.36–0.51)	0.72 (0.66–0.79)	<0.001
	Model1b	1	0.74 (0.63–0.87)	0.67 (0.56–0.81)	0.65 (0.54–0.77)	0.55 (0.45–0.67)	0.82 (0.75–0.89)	<0.001
	Model2	1	0.74 (0.63–0.87)	0.67 (0.56–0.81)	0.65 (0.54–0.78)	0.56 (0.46–0.68)	0.82 (0.75–0.89)	<0.001
	Serum beta carotene (median, μmol/L)	0.11	0.17	0.23	0.32	0.54		
	Model1a	1	0.77 (0.64–0.91)	0.63 (0.54–0.74)	0.60 (0.51–0.69)	0.52 (0.44–0.62)	0.86 (0.81–0.92)	<0.001
	Model1b	1	0.81 (0.68–0.97)	0.70 (0.60–0.81)	0.71 (0.61–0.83)	0.65 (0.55–0.78)	0.93 (0.88–0.98)	<0.001
	Model2	1	0.81 (0.68–0.97)	0.71 (0.61–0.82)	0.72 (0.62–0.84)	0.66 (0.56–0.79)	0.94 (0.89–0.99)	<0.001
	Serum lycopene (median, μmol/L)	0.27	0.36	0.43	0.48	0.53		
	Model1a	1	0.84 (0.74–0.95)	0.66 (0.56–0.79)	0.67 (0.59–0.77)	0.65 (0.56–0.76)	0.86 (0.81–0.91)	<0.001
	Model1b	1	0.89 (0.78–1.02)	0.74 (0.62–0.87)	0.76 (0.67–0.86)	0.76 (0.65–0.89)	0.91 (0.86–0.96)	<0.001
	Model2	1	0.89 (0.78–1.02)	0.73 (0.62–0.87)	0.77 (0.68–0.87)	0.76 (0.65–0.88)	0.91 (0.86–0.96)	<0.001
	Serum lutein/zeaxanthin (median, μmol/L)	0.20	0.27	0.32	0.39	0.54		
	Model1a	1	0.80 (0.68–0.95)	0.71 (0.62–0.81)	0.64 (0.54–0.74)	0.62 (0.54–0.72)	0.89 (0.83–0.95)	<0.001
	Model1b	1	0.83 (0.69–1.00)	0.80 (0.69–0.93)	0.73 (0.62–0.85)	0.72 (0.62–0.84)	0.93 (0.88–0.99)	<0.001
	Model2	1	0.83 (0.69–1.00)	0.81 (0.70–0.94)	0.73 (0.62–0.86)	0.73 (0.63–0.85)	0.94 (0.88–1.00)	<0.001
	Serum beta cryptoxanthin (median, μmol/L)	0.06	0.09	0.12	0.15	0.24		
	Model1a	1	0.78 (0.69–0.89)	0.74 (0.63–0.87)	0.64 (0.55–0.74)	0.56 (0.50–0.64)	0.80 (0.75–0.84)	<0.001
	Model1b	1	0.88 (0.78–1.00)	0.86 (0.73–1.01)	0.78 (0.67–0.91)	0.74 (0.65–0.84)	0.89 (0.85–0.94)	<0.001
	Model2	1	0.88 (0.78–0.99)	0.86 (0.74–1.01)	0.78 (0.68–0.91)	0.74 (0.66–0.84)	0.90 (0.86–0.94)	<0.001
	Composite biomarker score (median)	−0.67	−0.36	−0.09	0.22	0.75		
	Total fruits and vegetables intake (median, times/month)	39.34	50.42	57.41	67.96	89.29		
	Model1a	1	0.72 (0.61–0.86)	0.60 (0.52–0.70)	0.52 (0.44–0.61)	0.44 (0.38–0.51)	0.77 (0.72–0.82)	<0.001
	Model1b	1	0.81 (0.68–0.97)	0.71 (0.61–0.83)	0.65 (0.55–0.76)	0.58 (0.50–0.68)	0.85 (0.80–0.90)	<0.001
	Model2	1	0.81 (0.68–0.97)	0.72 (0.61–0.84)	0.65 (0.55–0.77)	0.59 (0.50–0.69)	0.86 (0.81–0.91)	<0.001

The serum alpha–carotene concentration was significantly associated with the cancer mortality, with HRs (95% CIs) of 0.70 (0.54–0.90), 0.68 (0.48–0.95), 0.64 (0.43–0.95), and 0.44 (0.33–0.60) for comparisons of groups 2, 3, 4, and 5, respectively, with group 1 in model 2 (*P* < .001 for the trend; Table 2). The HRs (95% CIs) for the association between each one SD increase in serum alpha–carotene, lycopene, beta–cryptoxanthin concentrations, and composite biomarker score and cancer mortality in model 2 were 0.77 (0.66–0.89), 0.89 (0.81–0.98), 0.76 (0.65–0.89), and 0.79 (0.69–0.90), respectively (Table 2). The composite biomarker score was significantly associated with cancer mortality, with HRs (95% CIs) of 0.58 (0.44–0.77), 0.53 (0.38–0.74), and 0.44 (0.33–0.57) when comparing groups 3 to 5, respectively, with group 1 in model 2 (*P* < 0.001 for the trend; Table 2).

The relationships were not statistically significant between each one SD increase in serum vitamin C, carotenoids, and their composite biomarker score and CVD mortality, heart disease mortality, and cerebral disease mortality in model 2 (Table 2). Serum vitamin C concentration was inversely associated with all–cause mortality in model 2, with HRs (95% CIs) for each one SD of 0.91 (0.86–0.96). The HRs (95% CIs) for the association between each one SD increase in total carotenoid, serum alpha–carotene, beta–carotene, lycopene, lutein/zeaxanthin, and beta–cryptoxanthin concentrations and all–cause mortality were 0.89 (0.84–0.94), 0.82 (0.75–0.89), 0.94 (0.89–0.99), 0.91 (0.86–0.96), 0.94 (0.88–1.00), and 0.90 (0.86–0.94) in model 2, respectively. A higher composite biomarker score was associated with a decrease in all–cause mortality, with an HR (95% CI) of 0.86 (0.81–0.91) for each one SD increase in the score (Table 2). The HRs (95% CIs) for the association between the composite biomarker score and all–cause mortality were 0.81 (0.68–0.97), 0.72 (0.61–0.84), 0.65 (0.55–0.77), and 0.59 (0.50–0.69) when comparing groups 2, 3, 4, and 5, respectively, compared with the lowest concentrations (group 1) in model 2 (*P* < .001 for the trend; Table 2).

**Figure 2:**
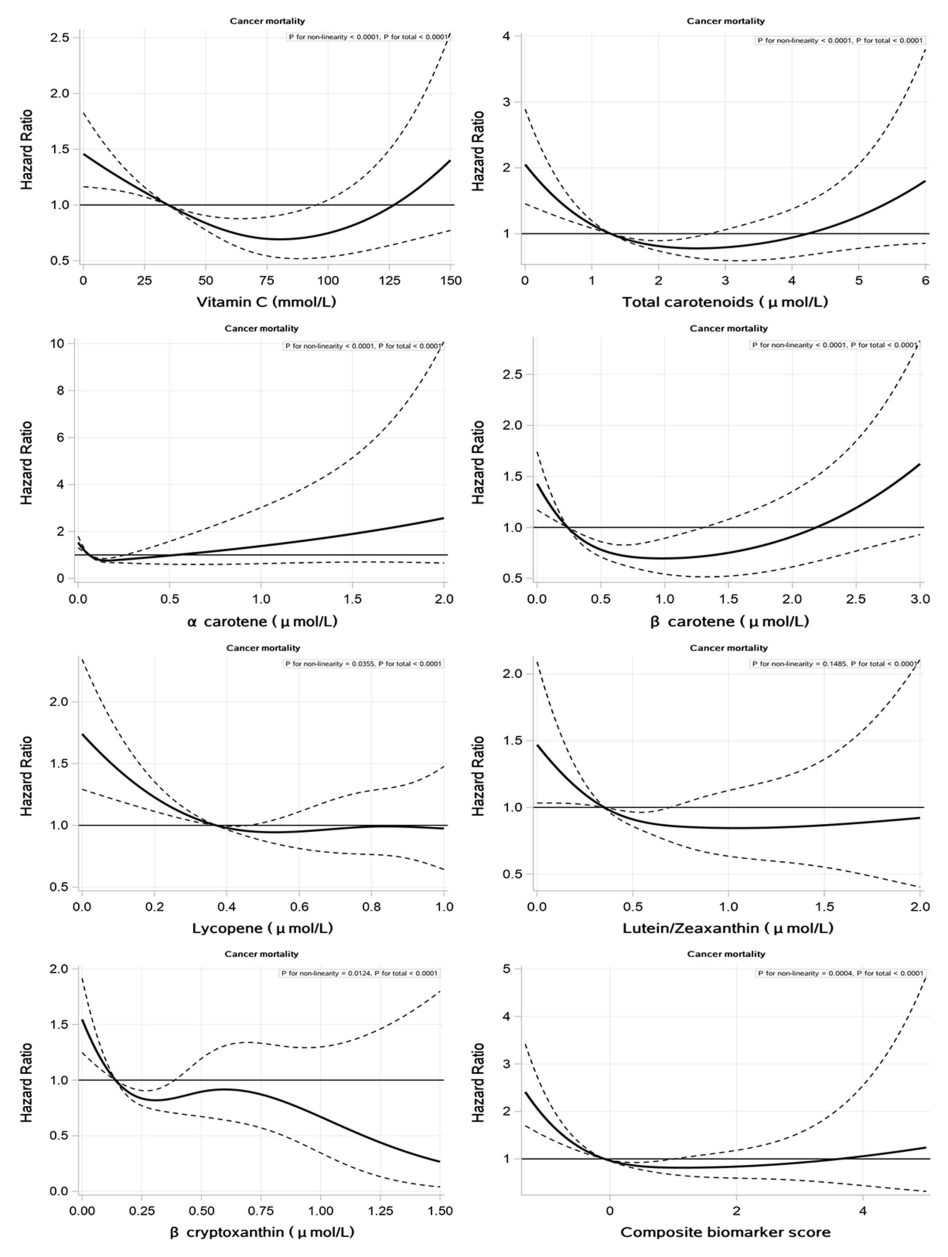
Associations of serum vitamin C, carotenoids, and composite biomarker scores with cancer mortality.

We found that non–linear associations were shown between cancer mortality and serum concentrations of vitamin C, total carotenoids and individual carotenoids, and composite biomarker score, expect for serum lutein/zeaxanthin (*P*–value for nonlinearity <0.05) (Figure 2). U–shaped relationships were found between serum vitamin C, total carotenoid concentrations, and composite biomarker score and cancer mortality (Figure 2). The null effect between very low and very high levels of serum vitamin C, total carotenoid concentrations, and composite biomarker score is shown in Figure 2. The figures of associations between serum biomarkers of fruit and vegetable intake and CVD, heart disease, cerebral disease, and all–cause mortality are shown in Supplementary Materials (Supplementary Figures 2–5).

### Sensitivity Analysis

The results did not change after excluding participants recruited within the first 2 years and the first 4 years and those with baseline cancer or CVD (Supplementary Table 3). The results also remain unchanged after further adjustments for hypertension, diabetes, and a history of cancer or CVD (Supplementary Table 3). Moreover, excluding one biomarker at a time to amend the composite biomarker score did not change the results (Supplementary Table 4). Furthermore, we calculated the E–value to evaluate the effect of unmeasured confounding between serum vitamin C concentration (E–value for each one SD, 1.81) and composite biomarker score (E–value for each one SD, 1.85) and cancer mortality. E–values for each one SD change in serum vitamin C concentration, total carotenoid concentration, and composite biomarker score with all–cause mortality were 1.43, 1.50, and 1.60, respectively (Supplementary Table 5).

## Discussion

In this prospective study, we found that inverse association between each one SD increases in serum vitamin C and cancer and all–cause mortality. Both the total carotenoids and the five individual carotenoids, expect for serum lutein/zeaxanthin, were negatively associated with all–cause mortality, whereas serum alpha–carotene, lycopene, and beta–cryptoxanthin concentrations were negatively associated with cancer mortality. Moreover, composite biomarker score was inversely associated with cancer mortality and all–cause mortality. The change for each one SD in the composite biomarker score, which is equivalent to an increase of approximately one time per quarter (0.483 times/month) in fruits and vegetables intake, the HR of cancer mortality was reduced by 21%. The relationships were not statistically significant between each one SD increase in serum vitamin C, carotenoids and their composite biomarker score and CVD, heart disease, and cerebral mortality.

A British study also shown an inverse association between serum vitamin C concentration and cancer mortality in older people (21). Vitamin C is a dietary antioxidant that can protect against oxidative stress and the harmful effects of reactive oxygen species (22). Consistent with our results, a meta–analysis showed a negative association between serum carotenoid concentration and cancer mortality (23). A positive association between the number of times per month that total fruits and vegetables were consumed and serum vitamin C was also observed in this study. Carotenoids are sources of various immune–protective substances and they are reported to have the protective effects on human health (24). Although studies of the individual carotenoids have given inconsistent results (6, 25). Serum carotenoids are powerful antioxidants that are important for the immune system (26). One of the most plentiful dietary carotenoids, alpha–carotene, inhibit the proliferation of human cancer cells (27). Similarly, a negative association between alpha–carotene levels and cancer mortality was found in this study. Previous studies have shown that root vegetable and carrot intake is a good proxy for serum alpha–carotene concentration (28). All of the above evidence indicates that strengthening the consumption of fruit and vegetable intake is beneficial to prevent cancer mortality.

In previous study of Americans, the relationship between vitamin C and all–cause mortality was consistent with this study (29). This study further explored the relationship between total carotenoids and individual carotenoids and all–cause mortality, as serum carotenoid levels are thought to reflect the intake of vegetables and fruits in an individual's diet (28, 30). Previous studies have found a negative association between serum total carotenoid, alpha–carotene, beta–carotene, lycopene, lutein/zeaxanthin, and beta–cryptoxanthin and all–cause mortality (24, 31, 32). Lycopene, a non–provitamin A carotenoid, has been shown to have direct anti–inflammatory effects (33, 34). Beta–carotene is a provitamin A carotenoid that contributes indirectly to the biological functions of vitamin A in human body and has been shown to have protective effects against chronic disease (35, 36).

We found that the composite biomarker score was negatively associated with cancer mortality and all–cause mortality, but it was not statistically significant with cerebral disease, heart disease, and CVD mortality. However, the association between participants with excessive composite biomarker score levels and cancer and all–cause mortality is viewed with caution, as a null effect of increased risk can be seen in the restricted cubic splines. The composite biomarker score allowed us to analyze individual serum biomarkers of fruit and vegetable intake using a minimum number of variables (37). A previous randomized controlled trial also found that a combined biomarker approach may be better than a single biomarker at determining the effect of a mixed fruit and vegetable diet (38). Also, the WHO encourages an increase intake of fruits and vegetables and recommends at least five servings (~400 g) of fruit or vegetables per day (39). Therefore, our findings support the intake of fruits and vegetables together.

The strengths of this study include the analysis of objective serum biomarkers of fruit and vegetable intake (serum vitamin C and carotenoid concentrations and their composite biomarker scores) with heart, CVD, cerebral, cancer, and all–cause mortality for the first time in a nationally representative sample of US population. Furthermore, we performed a number of sensitivity analyses, which showed robust findings, by excluding participants or adjusting for additional variables. Meanwhile, we quantified the potential effects of unmeasured confounders by the E–value sensitivity analysis and found that the results were unlikely affected by unmeasured confounders. However, this study also has some limitations. First, we were not able to classify the specific types of cancer due to limitations of the available data. Second, this study only analyzed serum biomarkers of fruit and vegetable intake in the general US population, which may limit the applicability of these findings to other populations. Finally, causality cannot be inferred because this study used an observational design.

## Conclusion

In this prospective study, inverse associations were found between serum vitamin C, carotenoids, and composite biomarker scores and outcomes expect for CVD, heart disease, and cerebral mortality in US adults. Our results indicate that the dietary intake of fruits and vegetables is crucial to promote public health, as it reduces the risk of all–cause mortality and cancer mortality.

## Data Availability Statement

Publicly available datasets were analyzed in this study. This data can be found here: the Third National Health and Nutrition Examination Survey, [NHANES III (1988–1994) (cdc.gov).

## Ethics Statement

The studies involving human participants were reviewed and approved by the National Health Statistics Research Ethics Review Board. The patients/participants provided their written informed consent to participate in this study.

## Author Contributions

LP: software, validation, and writing—original draft preparation. RZ: visualization and writing—reviewing. XW: supervision and writing—reviewing and editing. TZ: data curation and writing—reviewing. HS: methodology, software, and writing—reviewing and editing. LH: conceptualization, funding acquisition, and writing—reviewing and editing. The first draft of the manuscript was written by LP and all the authors commented on previous versions of the manuscript. All the authors have read and approved the final version of the manuscript.

## Funding

The study is supported by grants from the National Natural Science Foundation of China (Grant 82173648), Internal Fund of Ningbo Institute of Life and Health Industry, University of Chinese Academy of Sciences (2020YJY0212), the Public Welfare Foundation of Ningbo (2021S108), the Innovative Talent Support Plan of the Medical and Health Technology Project in Zhejiang province (2021422878), the Zhejiang Provincial Public Service and Application Research Foundation (LGF20H250001 and GC22H264267), Ningbo Health Branding Subject Fund (PPXK2018-01), and Sanming Project of Medicine in Shenzhen (SZSM201803080). The funders had no role in the design of the study or in the collection, analysis, and interpretation of data or in writing the manuscript.

## Conflict of Interest

The authors declare that the research was conducted in the absence of any commercial or financial relationships that could be construed as a potential conflict of interest.

## Publisher's Note

All claims expressed in this article are solely those of the authors and do not necessarily represent those of their affiliated organizations, or those of the publisher, the editors and the reviewers. Any product that may be evaluated in this article, or claim that may be made by its manufacturer, is not guaranteed or endorsed by the publisher.
