# Supplementary material for: Associations of Serum Biomarkers of Fruit and Vegetable Intake With the Risk of Cause–Specific Mortality and All–Cause Mortality: A National Prospective Cohort Study
Source: Front Nutr. 2022 May 11;9:874943. doi: 10.3389/fnut.2022.874943 (PMC9134271; doi:10.3389/fnut.2022.874943)
Supplement: Supplementary file 1 [file Data_Sheet_1.doc]

Supplementary materials

Table S1 Spearman correlation between serum vitamin C and carotenoids

| Variable | Vitamin C | Total carotenoids | Alpha carotene | Beta carotene | Lycopene | Lutein/  zeaxanthin | Beta cryptoxanthin | Composite biomarker score |
| --- | --- | --- | --- | --- | --- | --- | --- | --- |
| Vitamin C | 1 |  |  |  |  |  |  |  |
| Total carotenoids | 0.338 | 1 |  |  |  |  |  |  |
|  | <0.001 |  |  |  |  |  |  |  |
| Alpha carotene | 0.288 | 0.650 | 1 |  |  |  |  |  |
|  | <0.001 | <0.001 |  |  |  |  |  |  |
| Beta carotene | 0.301 | 0.819 | 0.640 | 1 |  |  |  |  |
|  | <0.001 | <0.001 | <0.001 |  |  |  |  |  |
| Lycopene | 0.030 | 0.510 | 0.106 | 0.147 | 1 |  |  |  |
|  | 0.001 | <0.001 | <0.001 | <0.001 |  |  |  |  |
| Lutein/zeaxanthin | 0.249 | 0.703 | 0.339 | 0.400 | 0.159 | 1 |  |  |
|  | <0.001 | <0.001 | <0.001 | <0.001 | <0.001 |  |  |  |
| Beta cryptoxanthin | 0.356 | 0.654 | 0.371 | 0.442 | 0.178 | 0.449 | 1 |  |
|  | <0.001 | <0.001 | <0.001 | <0.001 | <0.001 | <0.001 |  |  |
| Composite biomarker score | 0.590 | 0.950 | 0.703 | 0.751 | 0.436 | 0.678 | 0.704 | 1 |
|  | <0.001 | <0.001 | <0.001 | <0.001 | <0.001 | <0.001 | <0.001 |  |
| Vitamin A | 0.053 |  |  |  |  |  |  |  |
|  | <0.001 |  |  |  |  |  |  |  |
| Vitamin B12 | 0.007 |  |  |  |  |  |  |  |
|  | 0.562 |  |  |  |  |  |  |  |
| Vitamin E | 0.185 |  |  |  |  |  |  |  |
|  | <0.001 |  |  |  |  |  |  |  |

Table S2 Associations of demographic, lifestyle and dietary factors with serum vitamin C and carotenoids

| Variable | Serum vitamin C | | Total caroteneoids | | Alpha carotene | | Beta carotene | | Lycopene | | Lutein/zeaxanthin | | Beta cryptoxanthin | | Composite biomarker score | |
| --- | --- | --- | --- | --- | --- | --- | --- | --- | --- | --- | --- | --- | --- | --- | --- | --- |
|  | beta*(P)* | | beta*(P)* | | beta*(P)* | | beta*(P)* | | beta*(P)* | | beta*(P)* | | beta*(P)* | | beta*(P)* | |
| Age (year) | |  |  |  |  |  |  |  |  |  |  |  |  |  |  |  |
|  | 0.036 | 0.092 | 0.078 | 0.003 | 0.064 | <0.001 | 0.194 | <0.001 | -0.205 | <0.001 | 0.159 | <0.001 | 0.005 | 0.814 | 0.065 | 0.006 |
| Sex |  |  |  |  |  |  |  |  |  |  |  |  |  |  |  |  |
|  | 0.034 | 0.149 | -0.036 | 0.053 | 0.023 | 0.285 | 0.075 | <0.001 | -0.120 | <0.001 | -0.071 | 0.001 | -0.036 | 0.040 | -0.031 | 0.093 |
| Physical activity | | |  |  |  |  |  |  |  |  |  |  |  |  |  |  |
|  | 0.062 | 0.002 | 0.018 | 0.365 | 0.007 | 0.754 | 0.019 | 0.294 | 0.020 | 0.321 | 0.011 | 0.639 | -0.010 | 0.602 | 0.038 | 0.086 |
| Smoking status | | | |  |  |  |  |  |  |  |  |  |  |  |  |  |
|  | -0.182 | <0.001 | -0.164 | <0.001 | -0.136 | <0.001 | -0.127 | <0.001 | -0.037 | 0.180 | -0.125 | <0.001 | -0.180 | <0.001 | -0.197 | <0.001 |
| Marry status | | |  |  |  |  |  |  |  |  |  |  |  |  |  |  |
|  | -0.005 | 0.812 | -0.076 | 0.001 | -0.045 | 0.030 | -0.028 | 0.291 | -0.084 | <0.001 | -0.055 | 0.003 | -0.050 | 0.048 | -0.077 | <0.001 |
| Educational status | | |  |  |  |  |  |  |  |  |  |  |  |  |  |  |
|  | 0.052 | 0.027 | 0.061 | 0.006 | 0.061 | 0.008 | 0.040 | 0.039 | 0.104 | <0.001 | -0.011 | 0.690 | 0.013 | 0.458 | 0.071 | 0.002 |
| Alcohol consumption (times/week) | | | |  |  |  |  |  |  |  |  |  |  |  |  |  |
|  | -0.040 | 0.057 | -0.075 | 0.001 | -0.081 | <0.001 | -0.099 | <0.001 | -0.020 | 0.376 | -0.008 | 0.779 | -0.055 | 0.003 | -0.073 | 0.001 |
| Calories |  |  |  |  |  |  |  |  |  |  |  |  |  |  |  |  |
|  | 0.035 | 0.124 | 0.054 | 0.003 | 0.024 | 0.190 | 0.036 | 0.055 | 0.043 | 0.095 | 0.036 | 0.098 | 0.041 | 0.113 | 0.052 | 0.004 |
| HDL-C (mmol/L) | |  |  |  |  |  |  |  |  |  |  |  |  |  |  |  |
|  | 0.032 | 0.068 | 0.119 | <0.001 | 0.051 | 0.011 | 0.080 | 0.007 | 0.077 | 0.016 | 0.104 | <0.001 | 0.080 | <0.001 | 0.109 | <0.001 |
| BMI (kg/m^2^) | |  |  |  |  |  |  |  |  |  |  |  |  |  |  |  |
|  | -0.059 | 0.014 | -0.122 | <0.001 | -0.115 | <0.001 | -0.161 | <0.001 | 0.007 | 0.798 | -0.060 | <0.001 | -0.094 | <0.001 | -0.113 | <0.001 |
| Total fruits and vegetables (times/month) | | | |  |  |  |  |  |  |  |  |  |  |  |  |  |
|  | 0.283 | 0.029 | 0.165 | 0.259 | 0.078 | 0.415 | -0.162 | 0.141 | 0.347 | 0.055 | 0.287 | 0.034 | 0.042 | 0.744 | 0.282 | 0.042 |
| Citrus fruits (times/month) | | | |  |  |  |  |  |  |  |  |  |  |  |  |  |
|  | -0.013 | 0.686 | -0.013 | 0.657 | -0.027 | 0.308 | 0.041 | 0.104 | -0.114 | 0.003 | -0.047 | 0.153 | 0.152 | 0.001 | -0.021 | 0.469 |
| Non-citrus fruits (times/month) | | | |  |  |  |  |  |  |  |  |  |  |  |  |  |
|  | -0.036 | 0.518 | -0.008 | 0.914 | 0.064 | 0.241 | 0.139 | 0.024 | -0.143 | 0.052 | -0.097 | 0.106 | 0.024 | 0.701 | -0.026 | 0.697 |
| Green vegetables (times/month) | | | |  |  |  |  |  |  |  |  |  |  |  |  |  |
|  | -0.055 | 0.193 | 0.041 | 0.281 | -0.020 | 0.587 | 0.117 | 0.005 | -0.043 | 0.289 | 0.024 | 0.436 | -0.004 | 0.889 | 0.000 | 0.998 |
| Fruit_vegetables (times/month) | | | |  |  |  |  |  |  |  |  |  |  |  |  |  |
|  | -0.076 | 0.182 | 0.045 | 0.450 | -0.011 | 0.795 | 0.120 | 0.030 | -0.097 | 0.148 | 0.007 | 0.881 | 0.135 | 0.179 | -0.016 | 0.760 |
| Carrots (times/month) | | | |  |  |  |  |  |  |  |  |  |  |  |  |  |
|  | -0.052 | 0.037 | 0.068 | 0.085 | 0.258 | <0.001 | 0.167 | <0.001 | -0.087 | 0.014 | -0.035 | 0.215 | 0.003 | 0.930 | 0.057 | 0.140 |
| Broccoli (times/month) | | | |  |  |  |  |  |  |  |  |  |  |  |  |  |
|  | 0.083 | 0.145 | 0.018 | 0.657 | -0.036 | 0.428 | 0.018 | 0.515 | -0.029 | 0.423 | 0.067 | 0.183 | 0.012 | 0.739 | 0.030 | 0.519 |
| Cabbage, cole slaw, sauerkraut (times/month) | | | | |  |  |  |  |  |  |  |  |  |  |  |  |
|  | -0.018 | 0.651 | -0.032 | 0.209 | 0.020 | 0.475 | -0.047 | 0.090 | 0.010 | 0.804 | -0.047 | 0.109 | -0.008 | 0.762 | -0.030 | 0.278 |
| Any other vegetables (times/month) | | | |  |  |  |  |  |  |  |  |  |  |  |  |  |
|  | -0.057 | 0.172 | -0.036 | 0.480 | -0.034 | 0.216 | 0.045 | 0.219 | -0.074 | 0.183 | -0.051 | 0.317 | -0.047 | 0.228 | -0.064 | 0.204 |
| Potatoes (times/month) | | | |  |  |  |  |  |  |  |  |  |  |  |  |  |
|  | -0.024 | 0.156 | -0.058 | <0.001 | -0.068 | 0.001 | -0.056 | 0.001 | 0.033 | 0.209 | -0.060 | <0.001 | -0.078 | 0.001 | -0.060 | <0.001 |
| Cereal (times/month) | | | |  |  |  |  |  |  |  |  |  |  |  |  |  |
|  | -0.003 | 0.912 | -0.054 | 0.015 | -0.036 | 0.040 | -0.021 | 0.322 | -0.072 | 0.001 | -0.026 | 0.096 | -0.034 | 0.196 | -0.045 | 0.025 |
| Milk (times/month) | | | |  |  |  |  |  |  |  |  |  |  |  |  |  |
|  | -0.008 | 0.636 | -0.035 | 0.086 | -0.028 | 0.050 | -0.025 | 0.011 | -0.013 | 0.457 | -0.042 | 0.181 | -0.006 | 0.756 | -0.031 | 0.179 |
| Fruit_juices (times/month) | | | |  |  |  |  |  |  |  |  |  |  |  |  |  |
|  | 0.193 | <0.001 | 0.031 | 0.257 | -0.001 | 0.973 | 0.020 | 0.443 | -0.018 | 0.370 | 0.020 | 0.310 | 0.116 | 0.001 | 0.068 | 0.023 |
| Soft_drinks (times/month) | | | |  |  |  |  |  |  |  |  |  |  |  |  |  |
|  | -0.066 | 0.008 | -0.062 | <0.001 | -0.022 | 0.246 | -0.051 | 0.002 | -0.012 | 0.568 | -0.059 | <0.001 | -0.068 | <0.001 | -0.068 | 0.001 |
| Fish (times/month) | | | |  |  |  |  |  |  |  |  |  |  |  |  |  |
|  | -0.017 | 0.486 | 0.016 | 0.589 | 0.089 | 0.044 | 0.023 | 0.473 | -0.034 | 0.120 | 0.029 | 0.328 | -0.021 | 0.383 | 0.013 | 0.620 |
| Red_meat (times/month) | | | |  |  |  |  |  |  |  |  |  |  |  |  |  |
|  | -0.039 | 0.140 | -0.046 | 0.116 | -0.055 | 0.067 | -0.036 | 0.214 | -0.003 | 0.821 | -0.046 | 0.064 | -0.033 | 0.247 | -0.051 | 0.116 |
| Legumes (times/month) | | | |  |  |  |  |  |  |  |  |  |  |  |  |  |
|  | 0.002 | 0.962 | -0.006 | 0.794 | 0.029 | 0.256 | -0.011 | 0.664 | -0.017 | 0.470 | -0.017 | 0.448 | 0.033 | 0.440 | 0.014 | 0.652 |
| Eggs (times/month) | | | |  |  |  |  |  |  |  |  |  |  |  |  |  |
|  | -0.022 | 0.416 | -0.003 | 0.882 | 0.012 | 0.645 | -0.005 | 0.803 | -0.080 | <0.001 | 0.050 | 0.003 | 0.046 | 0.189 | -0.005 | 0.819 |
| Peanuts (times/month) | | | | |  |  |  |  |  |  |  |  |  |  |  |  |
|  | 0.009 | 0.662 | -0.005 | 0.717 | -0.001 | 0.947 | 0.025 | 0.103 | -0.009 | 0.611 | -0.028 | 0.184 | -0.019 | 0.247 | -0.009 | 0.585 |
| Offals (times/month) | | | |  |  |  |  |  |  |  |  |  |  |  |  |  |
|  | -0.043 | 0.014 | -0.008 | 0.612 | -0.025 | 0.324 | -0.009 | 0.602 | -0.044 | 0.025 | 0.057 | 0.102 | -0.023 | 0.069 | -0.021 | 0.237 |

Table S3 Sensitivity analysis for the association between serum biomarkers of and cause-specific and all-cause mortality

| **All-cause** **and cause-specific** **mortality** | **Biomarkers** | **model** | **group 1** | **group 2** | **group 3** | **group 4** | **group 5** | **For each one standard deviation** | ***P* value for trend** |
| --- | --- | --- | --- | --- | --- | --- | --- | --- | --- |
| CVD Mortality | Vitamin C | Model 2 excluding first 2 years | 1.0 (ref) | 0.64 (0.52 - 0.80) | 0.70 (0.55 - 0.90) | 0.60 (0.46 - 0.79) | 0.66 (0.50 - 0.86) | 0.94 (0.86 - 1.03) | 0.007 |
|  | Total carotenoids | Model 2 excluding first 2 years | 1.0 (ref) | 0.89 (0.66 - 1.19) | 0.90 (0.69 - 1.17) | 1.02 (0.80 - 1.30) | 0.96 (0.73 - 1.25) | 0.99 (0.88 - 1.10) | 0.864 |
|  | Alpha carotene | Model 2 excluding first 2 years | 1.0 (ref) | 0.79 (0.62 - 1.00) | 0.84 (0.65 - 1.08) | 0.83 (0.62 - 1.10) | 0.77 (0.56 - 1.07) | 0.90 (0.80 - 1.02) | 0.256 |
|  | Beta carotene | Model 2 excluding first 2 years | 1.0 (ref) | 0.78 (0.57 - 1.08) | 0.72 (0.53 - 0.99) | 0.79 (0.60 - 1.03) | 0.77 (0.56 - 1.06) | 0.96 (0.89 - 1.03) | 0.235 |
|  | Lycopene | Model 2 excluding first 2 years | 1.0 (ref) | 1.05 (0.84 - 1.32) | 0.84 (0.65 - 1.10) | 0.99 (0.75 - 1.29) | 0.98 (0.75 - 1.27) | 1.04 (0.93 - 1.17) | 0.613 |
|  | Lutein/zeaxanthin | Model 2 excluding first 2 years | 1.0 (ref) | 0.77 (0.60 - 1.01) | 1.02 (0.78 - 1.33) | 0.85 (0.64 - 1.14) | 1.02 (0.77 - 1.36) | 1.03 (0.92 - 1.15) | 0.504 |
|  | Beta cryptoxanthin | Model 2 excluding first 2 years | 1.0 (ref) | 1.06 (0.82 - 1.37) | 0.94 (0.71 - 1.25) | 0.92 (0.71 - 1.19) | 0.87 (0.68 - 1.12) | 0.97 (0.88 - 1.06) | 0.265 |
|  | Composite biomarker score | Model 2 excluding first 2 years | 1.0 (ref) | 0.92 (0.67 - 1.26) | 0.94 (0.75 - 1.16) | 0.88 (0.64 - 1.22) | 0.81 (0.61 - 1.09) | 0.96 (0.85 - 1.07) | 0.173 |
| Heart Mortality | Vitamin C | Model 2 excluding first 2 years | 1.0 (ref) | 0.59 (0.45 - 0.76) | 0.68 (0.51 - 0.92) | 0.55 (0.40 - 0.77) | 0.64 (0.47 - 0.87) | 0.94 (0.83 - 1.05) | 0.010 |
|  | Total carotenoids | Model 2 excluding first 2 years | 1.0 (ref) | 0.93 (0.69 - 1.26) | 0.87 (0.65 - 1.18) | 1.09 (0.80 - 1.49) | 0.96 (0.70 - 1.32) | 1.01 (0.88 - 1.15) | 0.820 |
|  | Alpha carotene | Model 2 excluding first 2 years | 1.0 (ref) | 0.80 (0.61 - 1.07) | 0.82 (0.59 - 1.15) | 0.8 (0.56 - 1.14) | 0.81 (0.55 - 1.20) | 0.90 (0.78 - 1.04) | 0.359 |
|  | Beta carotene | Model 2 excluding first 2 years | 1.0 (ref) | 0.82 (0.58 - 1.18) | 0.82 (0.57 - 1.17) | 0.81 (0.59 - 1.11) | 0.81 (0.57 - 1.15) | 0.97 (0.88 - 1.07) | 0.311 |
|  | Lycopene | Model 2 excluding first 2 years | 1.0 (ref) | 1.10 (0.84 - 1.45) | 0.88 (0.66 - 1.18) | 1.04 (0.76 - 1.42) | 1.08 (0.78 - 1.50) | 1.08 (0.93 - 1.26) | 0.831 |
|  | Lutein/zeaxanthin | Model 2 excluding first 2 years | 1.0 (ref) | 0.74 (0.59 - 0.94) | 1.03 (0.78 - 1.38) | 0.87 (0.68 - 1.11) | 1.02 (0.73 - 1.43) | 1.04 (0.92 - 1.19) | 0.493 |
|  | Beta cryptoxanthin | Model 2 excluding first 2 years | 1.0 (ref) | 1.11 (0.84 - 1.48) | 1.10 (0.80 - 1.50) | 0.95 (0.72 - 1.25) | 0.83 (0.61 - 1.12) | 0.94 (0.84 - 1.06) | 0.258 |
|  | Composite biomarker score | Model 2 excluding first 2 years | 1.0 (ref) | 0.92 (0.63 - 1.32) | 0.99 (0.72 - 1.36) | 0.93 (0.66 - 1.32) | 0.82 (0.58 - 1.16) | 0.97 (0.85 - 1.11) | 0.320 |
| Cerebral Mortality | Vitamin C | Model 2 excluding first 2 years | 1.0 (ref) | 0.97 (0.44 - 2.13) | 0.84 (0.47 - 1.52) | 0.87 (0.40 - 1.89) | 0.79 (0.44 - 1.44) | 0.96 (0.80 - 1.15) | 0.402 |
|  | Total carotenoids | Model 2 excluding first 2 years | 1.0 (ref) | 0.74 (0.36 - 1.53) | 1.00 (0.50 - 1.98) | 0.82 (0.44 - 1.50) | 0.93 (0.45 - 1.94) | 0.93 (0.80 - 1.08) | 0.947 |
|  | Alpha carotene | Model 2 excluding first 2 years | 1.0 (ref) | 0.72 (0.36 - 1.43) | 0.93 (0.53 - 1.63) | 0.95 (0.52 - 1.74) | 0.66 (0.30 - 1.48) | 0.89 (0.68 - 1.15) | 0.537 |
|  | Beta carotene | Model 2 excluding first 2 years | 1.0 (ref) | 0.62 (0.24 - 1.58) | 0.42 (0.20 - 0.89) | 0.71 (0.37 - 1.36) | 0.63 (0.29 - 1.37) | 0.93 (0.81 - 1.08) | 0.507 |
|  | Lycopene | Model 2 excluding first 2 years | 1.0 (ref) | 0.90 (0.54 - 1.49) | 0.72 (0.44 - 1.17) | 0.84 (0.47 - 1.49) | 0.66 (0.35 - 1.24) | 0.89 (0.71 - 1.12) | 0.200 |
|  | Lutein/zeaxanthin | Model 2 excluding first 2 years | 1.0 (ref) | 0.91 (0.37 - 2.23) | 0.95 (0.48 - 1.90) | 0.81 (0.36 - 1.84) | 1.03 (0.52 - 2.04) | 0.97 (0.86 - 1.10) | 0.946 |
|  | Beta cryptoxanthin | Model 2 excluding first 2 years | 1.0 (ref) | 0.87 (0.46 - 1.67) | 0.47 (0.23 - 0.94) | 0.85 (0.5 - 1.46) | 0.94 (0.55 - 1.61) | 1.05 (0.90 - 1.21) | 0.686 |
|  | Composite biomarker score | Model 2 excluding first 2 years | 1.0 (ref) | 0.93 (0.45 - 1.90) | 0.74 (0.34 - 1.60) | 0.74 (0.38 - 1.41) | 0.77 (0.35 - 1.68) | 0.91 (0.76 - 1.08) | 0.363 |
| Cancer Mortality | Vitamin C | Model 2 excluding first 2 years | 1.0 (ref) | 0.87 (0.66 - 1.14) | 0.68 (0.48 - 0.95) | 0.53 (0.36 - 0.77) | 0.57 (0.40 - 0.80) | 0.81 (0.71 - 0.92) | 0.001 |
|  | Total carotenoids | Model 2 excluding first 2 years | 1.0 (ref) | 0.88 (0.58 - 1.33) | 0.87 (0.61 - 1.23) | 0.68 (0.49 - 0.94) | 0.64 (0.47 - 0.89) | 0.88 (0.76 - 1.03) | 0.001 |
|  | Alpha carotene | Model 2 excluding first 2 years | 1.0 (ref) | 0.70 (0.54 - 0.90) | 0.69 (0.50 - 0.96) | 0.67 (0.45 - 1.00) | 0.48 (0.36 - 0.64) | 0.78 (0.67 - 0.90) | <0.001 |
|  | Beta carotene | Model 2 excluding first 2 years | 1.0 (ref) | 0.82 (0.59 - 1.14) | 0.72 (0.51 - 1.02) | 0.74 (0.55 - 1.00) | 0.58 (0.42 - 0.78) | 0.93 (0.79 - 1.09) | 0.001 |
|  | Lycopene | Model 2 excluding first 2 years | 1.0 (ref) | 1.00 (0.72 - 1.38) | 0.70 (0.52 - 0.95) | 0.73 (0.53 - 1.00) | 0.87 (0.64 - 1.17) | 0.91 (0.83 - 1.01) | 0.087 |
|  | Lutein/zeaxanthin | Model 2 excluding first 2 years | 1.0 (ref) | 0.88 (0.62 - 1.25) | 0.71 (0.49 - 1.04) | 0.78 (0.54 - 1.14) | 0.69 (0.52 - 0.93) | 0.99 (0.82 - 1.18) | 0.013 |
|  | Beta cryptoxanthin | Model 2 excluding first 2 years | 1.0 (ref) | 0.91 (0.72 - 1.15) | 0.73 (0.54 - 0.99) | 0.78 (0.58 - 1.05) | 0.59 (0.43 - 0.82) | 0.78 (0.67 - 0.92) | 0.001 |
|  | Composite biomarker score | Model 2 excluding first 2 years | 1.0 (ref) | 0.75 (0.53 - 1.06) | 0.60 (0.45 - 0.81) | 0.56 (0.40 - 0.80) | 0.47 (0.36 - 0.62) | 0.81 (0.71 - 0.93) | <0.001 |
| All-cause Mortality | Vitamin C | Model 2 excluding first 2 years | 1.0 (ref) | 0.85 (0.75 - 0.97) | 0.78 (0.67 - 0.90) | 0.73 (0.62 - 0.85) | 0.72 (0.61 - 0.84) | 0.91 (0.86 - 0.96) | <0.001 |
|  | Total carotenoids | Model 2 excluding first 2 years | 1.0 (ref) | 0.84 (0.75 - 0.94) | 0.78 (0.67 - 0.91) | 0.74 (0.64 - 0.86) | 0.67 (0.58 - 0.77) | 0.89 (0.84 - 0.94) | <0.001 |
|  | Alpha carotene | Model 2 excluding first 2 years | 1.0 (ref) | 0.72 (0.62 - 0.85) | 0.67 (0.56 - 0.80) | 0.66 (0.55 - 0.78) | 0.56 (0.46 - 0.68) | 0.82 (0.76 - 0.89) | <0.001 |
|  | Beta carotene | Model 2 excluding first 2 years | 1.0 (ref) | 0.82 (0.69 - 0.97) | 0.70 (0.60 - 0.81) | 0.72 (0.62 - 0.84) | 0.67 (0.56 - 0.79) | 0.94 (0.89 - 0.99) | <0.001 |
|  | Lycopene | Model 2 excluding first 2 years | 1.0 (ref) | 0.89 (0.78 - 1.03) | 0.74 (0.62 - 0.87) | 0.79 (0.70 - 0.89) | 0.76 (0.65 - 0.89) | 0.91 (0.86 - 0.97) | <0.001 |
|  | Lutein/zeaxanthin | Model 2 excluding first 2 years | 1.0 (ref) | 0.83 (0.68 - 1.00) | 0.81 (0.69 - 0.95) | 0.72 (0.61 - 0.86) | 0.72 (0.61 - 0.84) | 0.93 (0.87 - 0.99) | <0.001 |
|  | Beta cryptoxanthin | Model 2 excluding first 2 years | 1.0 (ref) | 0.88 (0.78 - 1.00) | 0.84 (0.72 - 0.99) | 0.77 (0.67 - 0.90) | 0.74 (0.65 - 0.83) | 0.90 (0.86 - 0.94) | <0.001 |
|  | Composite biomarker score | Model 2 excluding first 2 years | 1.0 (ref) | 0.82 (0.68 - 0.98) | 0.72 (0.62 - 0.85) | 0.65 (0.55 - 0.77) | 0.60 (0.51 - 0.70) | 0.86 (0.81 - 0.91) | <0.001 |
| CVD Mortality | Vitamin C | Model 2 excluding first 4 years | 1.0 (ref) | 0.62 (0.49 - 0.77) | 0.67 (0.51 - 0.86) | 0.61 (0.47 - 0.80) | 0.68 (0.52 - 0.88) | 0.96 (0.86 - 1.06) | 0.024 |
|  | Total carotenoids | Model 2 excluding first 4 years | 1.0 (ref) | 0.82 (0.61 - 1.09) | 0.89 (0.67 - 1.17) | 1.00 (0.78 - 1.28) | 0.93 (0.69 - 1.26) | 0.98 (0.87 - 1.11) | 0.886 |
|  | Alpha carotene | Model 2 excluding first 4 years | 1.0 (ref) | 0.79 (0.61 - 1.01) | 0.84 (0.67 - 1.05) | 0.79 (0.59 - 1.06) | 0.78 (0.56 - 1.08) | 0.88 (0.78 - 1.00) | 0.241 |
|  | Beta carotene | Model 2 excluding first 4 years | 1.0 (ref) | 0.77 (0.53 - 1.10) | 0.70 (0.52 - 0.94) | 0.78 (0.60 - 1.03) | 0.75 (0.53 - 1.06) | 0.94 (0.87 - 1.03) | 0.203 |
|  | Lycopene | Model 2 excluding first 4 years | 1.0 (ref) | 1.05 (0.82 - 1.34) | 0.80 (0.60 - 1.08) | 0.97 (0.72 - 1.30) | 0.98 (0.74 - 1.31) | 1.05 (0.92 - 1.19) | 0.620 |
|  | Lutein/zeaxanthin | Model 2 excluding first 4 years | 1.0 (ref) | 0.75 (0.56 - 1.00) | 1.04 (0.78 - 1.37) | 0.84 (0.61 - 1.14) | 0.99 (0.73 - 1.34) | 1.03 (0.91 - 1.16) | 0.644 |
|  | Beta cryptoxanthin | Model 2 excluding first 4 years | 1.0 (ref) | 1.05 (0.81 - 1.36) | 0.96 (0.71 - 1.31) | 0.89 (0.68 - 1.17) | 0.9 (0.67 - 1.21) | 0.97 (0.88 - 1.08) | 0.332 |
|  | Composite biomarker score | Model 2 excluding first 4 years | 1.0 (ref) | 0.92 (0.65 - 1.30) | 0.92 (0.73 - 1.16) | 0.89 (0.66 - 1.20) | 0.83 (0.60 - 1.15) | 0.96 (0.85 - 1.09) | 0.255 |
| Heart Mortality | Vitamin C | Model 2 excluding first 4 years | 1.0 (ref) | 0.56 (0.43 - 0.73) | 0.67 (0.49 - 0.92) | 0.58 (0.42 - 0.81) | 0.66 (0.49 - 0.89) | 0.95 (0.84 - 1.07) | 0.031 |
|  | Total carotenoids | Model 2 excluding first 4 years | 1.0 (ref) | 0.86 (0.63 - 1.17) | 0.88 (0.64 - 1.22) | 1.07 (0.78 - 1.47) | 0.97 (0.68 - 1.38) | 1.01 (0.88 - 1.17) | 0.709 |
|  | Alpha carotene | Model 2 excluding first 4 years | 1.0 (ref) | 0.82 (0.61 - 1.09) | 0.83 (0.60 - 1.14) | 0.74 (0.51 - 1.08) | 0.84 (0.56 - 1.26) | 0.89 (0.77 - 1.03) | 0.373 |
|  | Beta carotene | Model 2 excluding first 4 years | 1.0 (ref) | 0.81 (0.55 - 1.20) | 0.78 (0.55 - 1.11) | 0.79 (0.58 - 1.08) | 0.80 (0.55 - 1.17) | 0.96 (0.86 - 1.07) | 0.322 |
|  | Lycopene | Model 2 excluding first 4 years | 1.0 (ref) | 1.09 (0.81 - 1.46) | 0.82 (0.59 - 1.15) | 1.05 (0.75 - 1.46) | 1.11 (0.78 - 1.59) | 1.10 (0.93 - 1.30) | 0.726 |
|  | Lutein/zeaxanthin | Model 2 excluding first 4 years | 1.0 (ref) | 0.72 (0.55 - 0.93) | 1.04 (0.76 - 1.42) | 0.84 (0.65 - 1.09) | 1.00 (0.70 - 1.42) | 1.05 (0.91 - 1.20) | 0.590 |
|  | Beta cryptoxanthin | Model 2 excluding first 4 years | 1.0 (ref) | 1.12 (0.85 - 1.48) | 1.14 (0.81 - 1.61) | 0.93 (0.72 - 1.21) | 0.87 (0.62 - 1.23) | 0.95 (0.84 - 1.08) | 0.350 |
|  | Composite biomarker score | Model 2 excluding first 4 years | 1.0 (ref) | 0.96 (0.64 - 1.44) | 0.98 (0.69 - 1.41) | 0.95 (0.69 - 1.31) | 0.88 (0.6 - 1.29) | 0.98 (0.85 - 1.14) | 0.497 |
| Cerebral Mortality | Vitamin C | Model 2 excluding first 4 years | 1.0 (ref) | 0.93 (0.41 - 2.09) | 0.66 (0.35 - 1.26) | 0.75 (0.33 - 1.7) | 0.78 (0.42 - 1.47) | 0.98 (0.80 - 1.20) | 0.434 |
|  | Total carotenoids | Model 2 excluding first 4 years | 1.0 (ref) | 0.68 (0.32 - 1.44) | 0.88 (0.43 - 1.83) | 0.79 (0.41 - 1.53) | 0.79 (0.36 - 1.73) | 0.89 (0.75 - 1.05) | 0.688 |
|  | Alpha carotene | Model 2 excluding first 4 years | 1.0 (ref) | 0.68 (0.32 - 1.46) | 0.89 (0.50 - 1.60) | 0.97 (0.51 - 1.84) | 0.58 (0.25 - 1.32) | 0.86 (0.65 - 1.13) | 0.435 |
|  | Beta carotene | Model 2 excluding first 4 years | 1.0 (ref) | 0.61 (0.23 - 1.63) | 0.42 (0.20 - 0.89) | 0.74 (0.37 - 1.50) | 0.56 (0.25 - 1.27) | 0.88 (0.74 - 1.04) | 0.360 |
|  | Lycopene | Model 2 excluding first 4 years | 1.0 (ref) | 0.91 (0.52 - 1.59) | 0.73 (0.44 - 1.21) | 0.72 (0.38 - 1.39) | 0.60 (0.29 - 1.23) | 0.85 (0.66 - 1.11) | 0.116 |
|  | Lutein/zeaxanthin | Model 2 excluding first 4 years | 1.0 (ref) | 0.88 (0.35 - 2.19) | 1 .00 (0.48 - 2.08) | 0.83 (0.35 - 1.97) | 0.97 (0.46 - 2.05) | 0.97 (0.84 - 1.12) | 0.932 |
|  | Beta cryptoxanthin | Model 2 excluding first 4 years | 1.0 (ref) | 0.83 (0.43 - 1.63) | 0.43 (0.21 - 0.90) | 0.77 (0.42 - 1.42) | 0.93 (0.52 - 1.67) | 1.04 (0.88 - 1.23) | 0.600 |
|  | Composite biomarker score | Model 2 excluding first 4 years | 1.0 (ref) | 0.77 (0.36 - 1.69) | 0.71 (0.33 - 1.55) | 0.70 (0.36 - 1.37) | 0.66 (0.29 - 1.49) | 0.88 (0.72 - 1.06) | 0.280 |
| Cancer Mortality | Vitamin C | Model 2 excluding first 4 years | 1.0 (ref) | 0.81 (0.61 - 1.09) | 0.68 (0.48 - 0.94) | 0.53 (0.35 - 0.79) | 0.57 (0.40 - 0.80) | 0.83 (0.73 - 0.94) | 0.001 |
|  | Total carotenoids | Model 2 excluding first 4 years | 1.0 (ref) | 0.88 (0.58 - 1.35) | 0.87 (0.60 - 1.24) | 0.71 (0.49 - 1.03) | 0.64 (0.45 - 0.90) | 0.89 (0.76 - 1.05) | 0.003 |
|  | Alpha carotene | Model 2 excluding first 4 years | 1.0 (ref) | 0.68 (0.53 - 0.88) | 0.67 (0.48 - 0.93) | 0.70 (0.46 - 1.07) | 0.49 (0.35 - 0.68) | 0.80 (0.68 - 0.94) | 0.001 |
|  | Beta carotene | Model 2 excluding first 4 years | 1.0 (ref) | 0.85 (0.61 - 1.18) | 0.74 (0.52 - 1.04) | 0.79 (0.58 - 1.08) | 0.56 (0.40 - 0.78) | 0.92 (0.77 - 1.10) | 0.004 |
|  | Lycopene | Model 2 excluding first 4 years | 1.0 (ref) | 1.02 (0.72 - 1.46) | 0.73 (0.53 - 1.00) | 0.74 (0.54 - 1.02) | 0.89 (0.65 - 1.21) | 0.92 (0.83 - 1.01) | 0.119 |
|  | Lutein/zeaxanthin | Model 2 excluding first 4 years | 1.0 (ref) | 0.90 (0.63 - 1.27) | 0.71 (0.48 - 1.05) | 0.81 (0.55 - 1.21) | 0.69 (0.50 - 0.96) | 1.00 (0.83 - 1.21) | 0.033 |
|  | Beta cryptoxanthin | Model 2 excluding first 4 years | 1.0 (ref) | 0.86 (0.67 - 1.10) | 0.68 (0.48 - 0.96) | 0.81 (0.60 - 1.09) | 0.61 (0.44 - 0.84) | 0.80 (0.68 - 0.95) | 0.003 |
|  | Composite biomarker score | Model 2 excluding first 4 years | 1.0 (ref) | 0.70 (0.49 - 0.99) | 0.60 (0.44 - 0.81) | 0.57 (0.40 - 0.82) | 0.47 (0.35 - 0.64) | 0.83 (0.72 - 0.95) | <0.001 |
| All-cause Mortality | Vitamin C | Model 2 excluding first 4 years | 1.0 (ref) | 0.84 (0.73 - 0.97) | 0.77 (0.67 - 0.89) | 0.74 (0.63 - 0.87) | 0.72 (0.62 - 0.84) | 0.92 (0.87 - 0.97) | <0.001 |
|  | Total carotenoids | Model 2 excluding first 4 years | 1.0 (ref) | 0.83 (0.74 - 0.93) | 0.77 (0.66 - 0.89) | 0.75 (0.64 - 0.88) | 0.66 (0.57 - 0.77) | 0.89 (0.83 - 0.95) | <0.001 |
|  | Alpha carotene | Model 2 excluding first 4 years | 1.0 (ref) | 0.71 (0.61 - 0.83) | 0.66 (0.55 - 0.79) | 0.66 (0.55 - 0.79) | 0.55 (0.45 - 0.68) | 0.82 (0.76 - 0.90) | <0.001 |
|  | Beta carotene | Model 2 excluding first 4 years | 1.0 (ref) | 0.83 (0.69 - 1.00) | 0.70 (0.60 - 0.81) | 0.73 (0.62 - 0.86) | 0.66 (0.56 - 0.79) | 0.93 (0.88 - 0.99) | <0.001 |
|  | Lycopene | Model 2 excluding first 4 years | 1.0 (ref) | 0.90 (0.78 - 1.03) | 0.74 (0.62 - 0.87) | 0.80 (0.71 - 0.90) | 0.77 (0.65 - 0.90) | 0.92 (0.86 - 0.98) | <0.001 |
|  | Lutein/zeaxanthin | Model 2 excluding first 4 years | 1.0 (ref) | 0.83 (0.68 - 1.01) | 0.82 (0.70 - 0.96) | 0.73 (0.61 - 0.87) | 0.71 (0.59 - 0.84) | 0.93 (0.87 - 1.00) | <0.001 |
|  | Beta cryptoxanthin | Model 2 excluding first 4 years | 1.0 (ref) | 0.85 (0.74 - 0.98) | 0.84 (0.70 - 1.01) | 0.77 (0.67 - 0.90) | 0.73 (0.64 - 0.84) | 0.90 (0.86 - 0.94) | <0.001 |
|  | Composite biomarker score | Model 2 excluding first 4 years | 1.0 (ref) | 0.81 (0.67 - 0.98) | 0.71 (0.60 - 0.83) | 0.66 (0.56 - 0.78) | 0.60 (0.51 - 0.71) | 0.86 (0.81 - 0.92) | <0.001 |
| CVD Mortality | Vitamin C | Model 2 excluding baseline cancer and CVD cases | 1.0 (ref) | 0.61 (0.44 - 0.84) | 0.59 (0.42 - 0.84) | 0.52 (0.35 - 0.76) | 0.62 (0.47 - 0.83) | 0.93 (0.83 - 1.05) | 0.005 |
|  | Total carotenoids | Model 2 excluding baseline cancer and CVD cases | 1.0 (ref) | 0.89 (0.58 - 1.37) | 0.87 (0.60 - 1.26) | 0.93 (0.71 - 1.23) | 0.88 (0.63 - 1.22) | 0.96 (0.84 - 1.10) | 0.551 |
|  | Alpha carotene | Model 2 excluding baseline cancer and CVD cases | 1.0 (ref) | 0.70 (0.54 - 0.90) | 0.75 (0.57 - 0.99) | 0.78 (0.57 - 1.07) | 0.74 (0.54 - 1.01) | 0.88 (0.78 - 0.99) | 0.212 |
|  | Beta carotene | Model 2 excluding baseline cancer and CVD cases | 1.0 (ref) | 0.78 (0.52 - 1.16) | 0.69 (0.51 - 0.95) | 0.74 (0.55 - 1.01) | 0.73 (0.53 - 1.02) | 0.92 (0.83 - 1.02) | 0.108 |
|  | Lycopene | Model 2 excluding baseline cancer and CVD cases | 1.0 (ref) | 0.92 (0.68 - 1.23) | 0.70 (0.51 - 0.96) | 0.97 (0.69 - 1.35) | 0.85 (0.58 - 1.24) | 1.04 (0.88 - 1.22) | 0.363 |
|  | Lutein/zeaxanthin | Model 2 excluding baseline cancer and CVD cases | 1.0 (ref) | 0.81 (0.59 - 1.11) | 0.99 (0.71 - 1.38) | 0.76 (0.55 - 1.06) | 0.92 (0.66 - 1.30) | 1.01 (0.87 - 1.18) | 0.652 |
|  | Beta cryptoxanthin | Model 2 excluding baseline cancer and CVD cases | 1.0 (ref) | 1.00 (0.73 - 1.37) | 0.90 (0.58 - 1.37) | 0.95 (0.64 - 1.40) | 0.82 (0.58 - 1.15) | 0.96 (0.86 - 1.08) | 0.372 |
|  | Composite biomarker score | Model 2 excluding baseline cancer and CVD cases | 1.0 (ref) | 0.85 (0.57 - 1.27) | 0.88 (0.66 - 1.18) | 0.88 (0.61 - 1.27) | 0.75 (0.54 - 1.04) | 0.94 (0.82 - 1.07) | 0.142 |
| Heart Mortality | Vitamin C | Model 2 excluding baseline cancer and CVD cases | 1.0 (ref) | 0.54 (0.39 - 0.75) | 0.60 (0.42 - 0.86) | 0.51 (0.33 - 0.78) | 0.61 (0.43 - 0.87) | 0.94 (0.81 - 1.08) | 0.016 |
|  | Total carotenoids | Model 2 excluding baseline cancer and CVD cases | 1.0 (ref) | 1.00 (0.63 - 1.57) | 0.87 (0.59 - 1.29) | 1.07 (0.78 - 1.47) | 0.93 (0.62 - 1.37) | 0.99 (0.84 - 1.18) | 0.876 |
|  | Alpha carotene | Model 2 excluding baseline cancer and CVD cases | 1.0 (ref) | 0.73 (0.54 - 0.98) | 0.74 (0.51 - 1.07) | 0.73 (0.50 - 1.07) | 0.79 (0.54 - 1.17) | 0.87 (0.75 - 1.00) | 0.314 |
|  | Beta carotene | Model 2 excluding baseline cancer and CVD cases | 1.0 (ref) | 0.94 (0.60 - 1.48) | 0.88 (0.59 - 1.31) | 0.92 (0.64 - 1.32) | 0.84 (0.58 - 1.22) | 0.93 (0.80 - 1.07) | 0.379 |
|  | Lycopene | Model 2 excluding baseline cancer and CVD cases | 1.0 (ref) | 1.00 (0.72 - 1.40) | 0.83 (0.59 - 1.17) | 1.04 (0.74 - 1.46) | 1.09 (0.74 - 1.58) | 1.12 (0.93 - 1.34) | 0.657 |
|  | Lutein/zeaxanthin | Model 2 excluding baseline cancer and CVD cases | 1.0 (ref) | 0.78 (0.55 - 1.09) | 1.01 (0.74 - 1.39) | 0.81 (0.60 - 1.08) | 0.90 (0.61 - 1.35) | 1.03 (0.87 - 1.23) | 0.733 |
|  | Beta cryptoxanthin | Model 2 excluding baseline cancer and CVD cases | 1.0 (ref) | 1.08 (0.80 - 1.46) | 1.10 (0.68 - 1.79) | 0.91 (0.60 - 1.38) | 0.79 (0.53 - 1.18) | 0.92 (0.79 - 1.08) | 0.303 |
|  | Composite biomarker score | Model 2 excluding baseline cancer and CVD cases | 1.0 (ref) | 0.95 (0.61 - 1.47) | 1.00 (0.7 - 1.42) | 1.01 (0.70 - 1.45) | 0.79 (0.53 - 1.19) | 0.96 (0.82 - 1.13) | 0.359 |
| Cerebral Mortality | Vitamin C | Model 2 excluding baseline cancer and CVD cases | 1.0 (ref) | 0.92 (0.35 - 2.40) | 0.57 (0.26 - 1.27) | 0.57 (0.23 - 1.43) | 0.68 (0.32 - 1.46) | 0.92 (0.70 - 1.20) | 0.225 |
|  | Total carotenoids | Model 2 excluding baseline cancer and CVD cases | 1.0 (ref) | 0.58 (0.23 - 1.44) | 0.84 (0.36 - 1.95) | 0.55 (0.25 - 1.21) | 0.71 (0.30 - 1.67) | 0.87 (0.72 - 1.05) | 0.436 |
|  | Alpha carotene | Model 2 excluding baseline cancer and CVD cases | 1.0 (ref) | 0.59 (0.23 - 1.49) | 0.81 (0.41 - 1.57) | 0.93 (0.47 - 1.83) | 0.60 (0.25 - 1.44) | 0.91 (0.69 - 1.18) | 0.574 |
|  | Beta carotene | Model 2 excluding baseline cancer and CVD cases | 1.0 (ref) | 0.40 (0.14 - 1.15) | 0.27 (0.14 - 0.53) | 0.37 (0.19 - 0.69) | 0.46 (0.22 - 0.96) | 0.90 (0.76 - 1.06) | 0.125 |
|  | Lycopene | Model 2 excluding baseline cancer and CVD cases | 1.0 (ref) | 0.71 (0.35 - 1.43) | 0.37 (0.2 - 0.71) | 0.79 (0.41 - 1.55) | 0.30 (0.11 - 0.81) | 0.76 (0.54 - 1.07) | 0.032 |
|  | Lutein/zeaxanthin | Model 2 excluding baseline cancer and CVD cases | 1.0 (ref) | 0.92 (0.36 - 2.39) | 0.90 (0.39 - 2.10) | 0.63 (0.20 - 2.01) | 0.99 (0.47 - 2.09) | 0.94 (0.81 - 1.09) | 0.796 |
|  | Beta cryptoxanthin | Model 2 excluding baseline cancer and CVD cases | 1.0 (ref) | 0.73 (0.31 - 1.72) | 0.31 (0.14 - 0.71) | 1.04 (0.51 - 2.15) | 0.85 (0.43 - 1.68) | 1.07 (0.90 - 1.28) | 0.945 |
|  | Composite biomarker score | Model 2 excluding baseline cancer and CVD cases | 1.0 (ref) | 0.56 (0.23 - 1.37) | 0.56 (0.24 - 1.35) | 0.54 (0.24 - 1.21) | 0.58 (0.24 - 1.41) | 0.87 (0.69 - 1.09) | 0.281 |
| Cancer Mortality | Vitamin C | Model 2 excluding baseline cancer and CVD cases | 1.0 (ref) | 0.72 (0.51 - 1.02) | 0.59 (0.41 - 0.83) | 0.43 (0.27 - 0.71) | 0.52 (0.35 - 0.77) | 0.78 (0.68 - 0.89) | 0.001 |
|  | Total carotenoids | Model 2 excluding baseline cancer and CVD cases | 1.0 (ref) | 0.78 (0.50 - 1.23) | 0.78 (0.54 - 1.12) | 0.69 (0.48 - 1.00) | 0.56 (0.38 - 0.81) | 0.87 (0.72 - 1.06) | 0.001 |
|  | Alpha carotene | Model 2 excluding baseline cancer and CVD cases | 1.0 (ref) | 0.72 (0.55 - 0.93) | 0.72 (0.50 - 1.02) | 0.71 (0.47 - 1.09) | 0.45 (0.29 - 0.67) | 0.80 (0.67 - 0.96) | 0.001 |
|  | Beta carotene | Model 2 excluding baseline cancer and CVD cases | 1.0 (ref) | 0.92 (0.68 - 1.23) | 0.86 (0.59 - 1.25) | 0.74 (0.57 - 0.97) | 0.63 (0.46 - 0.85) | 0.94 (0.80 - 1.12) | 0.001 |
|  | Lycopene | Model 2 excluding baseline cancer and CVD cases | 1.0 (ref) | 0.83 (0.59 - 1.17) | 0.55 (0.41 - 0.72) | 0.58 (0.40 - 0.84) | 0.79 (0.53 - 1.17) | 0.87 (0.76 - 1.01) | 0.074 |
|  | Lutein/zeaxanthin | Model 2 excluding baseline cancer and CVD cases | 1.0 (ref) | 0.92 (0.64 - 1.32) | 0.7 (0.48 - 1.02) | 0.77 (0.52 - 1.12) | 0.67 (0.47 - 0.94) | 0.98 (0.79 - 1.22) | 0.011 |
|  | Beta cryptoxanthin | Model 2 excluding baseline cancer and CVD cases | 1.0 (ref) | 1.00 (0.79 - 1.25) | 0.69 (0.46 - 1.02) | 0.75 (0.55 - 1.01) | 0.62 (0.42 - 0.91) | 0.76 (0.62 - 0.94) | 0.001 |
|  | Composite biomarker score | Model 2 excluding baseline cancer and CVD cases | 1.0 (ref) | 0.68 (0.48 - 0.95) | 0.55 (0.39 - 0.76) | 0.52 (0.36 - 0.75) | 0.41 (0.28 - 0.6) | 0.78 (0.66 - 0.93) | <0.001 |
| All-cause Mortality | Vitamin C | Model 2 excluding baseline cancer and CVD cases | 1.0 (ref) | 0.77 (0.64 - 0.92) | 0.73 (0.63 - 0.85) | 0.66 (0.54 - 0.79) | 0.69 (0.58 - 0.81) | 0.90 (0.85 - 0.96) | <0.001 |
|  | Total carotenoids | Model 2 excluding baseline cancer and CVD cases | 1.0 (ref) | 0.83 (0.73 - 0.94) | 0.74 (0.61 - 0.88) | 0.70 (0.60 - 0.81) | 0.61 (0.50 - 0.74) | 0.87 (0.80 - 0.94) | <0.001 |
|  | Alpha carotene | Model 2 excluding baseline cancer and CVD cases | 1.0 (ref) | 0.71 (0.60 - 0.83) | 0.66 (0.56 - 0.79) | 0.64 (0.52 - 0.80) | 0.54 (0.43 - 0.67) | 0.81 (0.74 - 0.90) | <0.001 |
|  | Beta carotene | Model 2 excluding baseline cancer and CVD cases | 1.0 (ref) | 0.83 (0.68 - 1.00) | 0.70 (0.59 - 0.84) | 0.68 (0.57 - 0.81) | 0.64 (0.53 - 0.77) | 0.93 (0.88 - 0.98) | <0.001 |
|  | Lycopene | Model 2 excluding baseline cancer and CVD cases | 1.0 (ref) | 0.80 (0.68 - 0.93) | 0.66 (0.56 - 0.79) | 0.71 (0.60 - 0.83) | 0.69 (0.56 - 0.85) | 0.89 (0.82 - 0.97) | <0.001 |
|  | Lutein/zeaxanthin | Model 2 excluding baseline cancer and CVD cases | 1.0 (ref) | 0.86 (0.68 - 1.07) | 0.82 (0.69 - 0.96) | 0.69 (0.57 - 0.82) | 0.67 (0.55 - 0.80) | 0.91 (0.83 - 1.01) | <0.001 |
|  | Beta cryptoxanthin | Model 2 excluding baseline cancer and CVD cases | 1.0 (ref) | 0.88 (0.76 - 1.02) | 0.88 (0.75 - 1.04) | 0.76 (0.64 - 0.91) | 0.72 (0.61 - 0.84) | 0.88 (0.83 - 0.94) | <0.001 |
|  | Composite biomarker score | Model 2 excluding baseline cancer and CVD cases | 1.0 (ref) | 0.77 (0.63 - 0.94) | 0.68 (0.58 - 0.79) | 0.63 (0.52 - 0.76) | 0.54 (0.44 - 0.66) | 0.84 (0.77 - 0.91) | <0.001 |
| CVD Mortality | Vitamin C | Model 2 + other | 1.0 (ref) | 0.66 (0.54 - 0.81) | 0.68 (0.53 - 0.86) | 0.64 (0.49 - 0.84) | 0.66 (0.51 - 0.85) | 0.94 (0.86 - 1.03) | 0.009 |
|  | Total carotenoids | Model 2 + other | 1.0 (ref) | 0.85 (0.63 - 1.15) | 0.93 (0.72 - 1.20) | 1.04 (0.84 - 1.29) | 1.03 (0.81 - 1.30) | 1.01 (0.92 - 1.11) | 0.346 |
|  | Alpha carotene | Model 2 + other | 1.0 (ref) | 0.83 (0.65 - 1.06) | 0.88 (0.67 - 1.15) | 0.82 (0.63 - 1.06) | 0.85 (0.63 - 1.14) | 0.93 (0.83 - 1.03) | 0.367 |
|  | Beta carotene | Model 2 + other | 1.0 (ref) | 0.77 (0.57 - 1.04) | 0.74 (0.57 - 0.97) | 0.79 (0.65 - 0.97) | 0.84 (0.64 - 1.10) | 0.98 (0.92 - 1.04) | 0.434 |
|  | Lycopene | Model 2 + other | 1.0 (ref) | 1.10 (0.88 - 1.39) | 0.87 (0.67 - 1.12) | 0.98 (0.76 - 1.27) | 1.10 (0.87 - 1.39) | 1.06 (0.95 - 1.17) | 0.867 |
|  | Lutein/zeaxanthin | Model 2 + other | 1.0 (ref) | 0.76 (0.61 - 0.95) | 1.06 (0.85 - 1.33) | 0.89 (0.69 - 1.15) | 1.04 (0.82 - 1.32) | 1.04 (0.94 - 1.14) | 0.302 |
|  | Beta cryptoxanthin | Model 2 + other | 1.0 (ref) | 1.05 (0.82 - 1.35) | 0.97 (0.72 - 1.30) | 0.96 (0.75 - 1.24) | 0.92 (0.73 - 1.18) | 0.99 (0.91 - 1.09) | 0.512 |
|  | Composite biomarker score | Model 2 + other | 1.0 (ref) | 0.88 (0.65 - 1.19) | 0.96 (0.76 - 1.21) | 0.92 (0.69 - 1.23) | 0.88 (0.68 - 1.13) | 0.98 (0.90 - 1.08) | 0.418 |
| Heart Mortality | Vitamin C | Model 2 + other | 1.0 (ref) | 0.60 (0.47 - 0.77) | 0.66 (0.51 - 0.87) | 0.60 (0.44 - 0.81) | 0.64 (0.48 - 0.88) | 0.94 (0.84 - 1.06) | 0.017 |
|  | Total carotenoids | Model 2 + other | 1.0 (ref) | 0.89 (0.66 - 1.20) | 0.86 (0.65 - 1.14) | 1.10 (0.84 - 1.44) | 1.04 (0.80 - 1.36) | 1.04 (0.92 - 1.16) | 0.378 |
|  | Alpha carotene | Model 2 + other | 1.0 (ref) | 0.83 (0.63 - 1.09) | 0.87 (0.62 - 1.22) | 0.77 (0.55 - 1.07) | 0.88 (0.61 - 1.28) | 0.93 (0.81 - 1.06) | 0.475 |
|  | Beta carotene | Model 2 + other | 1.0 (ref) | 0.83 (0.60 - 1.15) | 0.85 (0.60 - 1.19) | 0.81 (0.63 - 1.06) | 0.89 (0.64 - 1.24) | 0.99 (0.91 - 1.07) | 0.619 |
|  | Lycopene | Model 2 + other | 1.0 (ref) | 1.16 (0.86 - 1.56) | 0.91 (0.68 - 1.20) | 1.00 (0.74 - 1.34) | 1.25 (0.95 - 1.65) | 1.10 (0.97 - 1.26) | 0.404 |
|  | Lutein/zeaxanthin | Model 2 + other | 1.0 (ref) | 0.71 (0.58 - 0.88) | 1.04 (0.82 - 1.31) | 0.90 (0.72 - 1.14) | 1.03 (0.77 - 1.37) | 1.06 (0.95 - 1.18) | 0.312 |
|  | Beta cryptoxanthin | Model 2 + other | 1.0 (ref) | 1.10 (0.84 - 1.43) | 1.12 (0.80 - 1.55) | 0.96 (0.73 - 1.25) | 0.89 (0.67 - 1.18) | 0.97 (0.86 - 1.08) | 0.421 |
|  | Composite biomarker score | Model 2 + other | 1.0 (ref) | 0.89 (0.62 - 1.26) | 1.00 (0.74 - 1.35) | 0.95 (0.70 - 1.29) | 0.90 (0.67 - 1.20) | 1.00 (0.90 - 1.12) | 0.650 |
| Cerebral Mortality | Vitamin C | Model 2 + other | 1.0 (ref) | 0.97 (0.47 - 2.02) | 0.77 (0.43 - 1.36) | 0.86 (0.41 - 1.82) | 0.77 (0.43 - 1.40) | 0.95 (0.79 - 1.14) | 0.355 |
|  | Total carotenoids | Model 2 + other | 1.0 (ref) | 0.72 (0.35 - 1.49) | 1.17 (0.60 - 2.30) | 0.86 (0.48 - 1.54) | 0.97 (0.48 - 1.99) | 0.94 (0.82 - 1.08) | 0.916 |
|  | Alpha carotene | Model 2 + other | 1.0 (ref) | 0.83 (0.43 - 1.62) | 0.96 (0.56 - 1.65) | 0.99 (0.54 - 1.80) | 0.73 (0.34 - 1.56) | 0.91 (0.71 - 1.17) | 0.589 |
|  | Beta carotene | Model 2 + other | 1.0 (ref) | 0.57 (0.24 - 1.39) | 0.40 (0.20 - 0.79) | 0.69 (0.37 - 1.26) | 0.65 (0.33 - 1.26) | 0.95 (0.83 - 1.07) | 0.540 |
|  | Lycopene | Model 2 + other | 1.0 (ref) | 0.94 (0.57 - 1.55) | 0.71 (0.43 - 1.16) | 0.92 (0.53 - 1.60) | 0.65 (0.34 - 1.26) | 0.89 (0.71 - 1.10) | 0.222 |
|  | Lutein/zeaxanthin | Model 2 + other | 1.0 (ref) | 0.96 (0.42 - 2.23) | 1.14 (0.58 - 2.24) | 0.83 (0.37 - 1.85) | 1.11 (0.58 - 2.10) | 0.97 (0.87 - 1.08) | 0.857 |
|  | Beta cryptoxanthin | Model 2 + other | 1.0 (ref) | 0.90 (0.48 - 1.68) | 0.48 (0.26 - 0.92) | 1.00 (0.60 - 1.67) | 0.99 (0.61 - 1.61) | 1.07 (0.93 - 1.24) | 0.986 |
|  | Composite biomarker score | Model 2 + other | 1.0 (ref) | 0.86 (0.44 - 1.70) | 0.83 (0.39 - 1.75) | 0.81 (0.43 - 1.53) | 0.79 (0.38 - 1.65) | 0.92 (0.78 - 1.08) | 0.470 |
| Cancer Mortality | Vitamin C | Model 2 + other | 1.0 (ref) | 0.83 (0.62 - 1.10) | 0.65 (0.48 - 0.89) | 0.51 (0.35 - 0.75) | 0.54 (0.38 - 0.75) | 0.80 (0.71 - 0.91) | <0.001 |
|  | Total carotenoids | Model 2 + other | 1.0 (ref) | 0.82 (0.56 - 1.20) | 0.85 (0.61 - 1.19) | 0.64 (0.47 - 0.88) | 0.60 (0.45 - 0.81) | 0.87 (0.75 - 1.01) | <0.001 |
|  | Alpha carotene | Model 2 + other | 1.0 (ref) | 0.70 (0.54 - 0.91) | 0.68 (0.48 - 0.96) | 0.64 (0.43 - 0.95) | 0.46 (0.33 - 0.63) | 0.78 (0.66 - 0.91) | <0.001 |
|  | Beta carotene | Model 2 + other | 1.0 (ref) | 0.82 (0.59 - 1.14) | 0.74 (0.53 - 1.03) | 0.73 (0.55 - 0.98) | 0.57 (0.42 - 0.77) | 0.92 (0.79 - 1.07) | 0.001 |
|  | Lycopene | Model 2 + other | 1.0 (ref) | 0.97 (0.72 - 1.30) | 0.67 (0.50 - 0.88) | 0.68 (0.50 - 0.94) | 0.83 (0.62 - 1.10) | 0.89 (0.81 - 0.98) | 0.028 |
|  | Lutein/zeaxanthin | Model 2 + other | 1.0 (ref) | 0.89 (0.62 - 1.26) | 0.71 (0.50 - 1.00) | 0.78 (0.54 - 1.14) | 0.70 (0.53 - 0.93) | 0.98 (0.82 - 1.17) | 0.011 |
|  | Beta cryptoxanthin | Model 2 + other | 1.0 (ref) | 0.90 (0.71 - 1.15) | 0.73 (0.54 - 0.99) | 0.75 (0.57 - 1.00) | 0.58 (0.43 - 0.79) | 0.77 (0.66 - 0.90) | <0.001 |
|  | Composite biomarker score | Model 2 + other | 1.0 (ref) | 0.70 (0.51 - 0.98) | 0.59 (0.44 - 0.79) | 0.53 (0.38 - 0.75) | 0.44 (0.34 - 0.57) | 0.80 (0.70 - 0.90) | <0.001 |
| All-cause Mortality | Vitamin C | Model 2 + other | 1.0 (ref) | 0.82 (0.72 - 0.94) | 0.76 (0.66 - 0.88) | 0.74 (0.64 - 0.86) | 0.71 (0.62 - 0.82) | 0.91 (0.87 - 0.96) | <0.001 |
|  | Total carotenoids | Model 2 + other | 1.0 (ref) | 0.81 (0.71 - 0.92) | 0.79 (0.68 - 0.91) | 0.74 (0.64 - 0.87) | 0.68 (0.59 - 0.78) | 0.90 (0.85 - 0.95) | <0.001 |
|  | Alpha carotene | Model 2 + other | 1.0 (ref) | 0.73 (0.63 - 0.86) | 0.67 (0.56 - 0.80) | 0.64 (0.54 - 0.77) | 0.57 (0.48 - 0.68) | 0.83 (0.77 - 0.90) | <0.001 |
|  | Beta carotene | Model 2 + other | 1.0 (ref) | 0.81 (0.68 - 0.95) | 0.71 (0.62 - 0.83) | 0.72 (0.63 - 0.83) | 0.69 (0.58 - 0.82) | 0.95 (0.90 - 1.00) | <0.001 |
|  | Lycopene | Model 2 + other | 1.0 (ref) | 0.91 (0.80 - 1.04) | 0.73 (0.61 - 0.88) | 0.77 (0.68 - 0.88) | 0.79 (0.68 - 0.92) | 0.91 (0.86 - 0.96) | <0.001 |
|  | Lutein/zeaxanthin | Model 2 + other | 1.0 (ref) | 0.82 (0.68 - 0.99) | 0.83 (0.72 - 0.96) | 0.73 (0.62 - 0.86) | 0.73 (0.64 - 0.84) | 0.94 (0.88 - 0.99) | <0.001 |
|  | Beta cryptoxanthin | Model 2 + other | 1.0 (ref) | 0.87 (0.77 - 0.98) | 0.84 (0.71 - 0.99) | 0.78 (0.67 - 0.89) | 0.76 (0.68 - 0.85) | 0.91 (0.87 - 0.95) | <0.001 |
|  | Composite biomarker score | Model 2 + other^*^ | 1.0 (ref) | 0.78 (0.65 - 0.93) | 0.73 (0.62 - 0.85) | 0.65 (0.55 - 0.76) | 0.60 (0.52 - 0.70) | 0.87 (0.82 - 0.92) | <0.001 |

^*^other shows that on the Model 2, disease-related variables are additionally controlled, including hypertension, diabetes, history of cancer and history of CVD

Table S4 Sensitivity analysis for the influence of individual biomarkers on the results of the composite biomarker score

| **y** | **model** | **group 1** | **group 2** | **group 3** | **group 4** | **group 5** | **For each one standard deviation** | ***P* value for trend** |
| --- | --- | --- | --- | --- | --- | --- | --- | --- |
| CVD Mortality | including all 6 biomarkers | 1.0 (ref) | 0.93 (0.69 - 1.25) | 0.94 (0.75 - 1.18) | 0.90 (0.66 - 1.22) | 0.84 (0.65 - 1.10) | 0.96 (0.87 - 1.06) | 0.220 |
| Heart Mortality |  | 1.0 (ref) | 0.93 (0.66 - 1.33) | 0.99 (0.73 - 1.34) | 0.94 (0.67 - 1.32) | 0.87 (0.64 - 1.18) | 0.98 (0.88 - 1.10) | 0.428 |
| Cerebral Mortality |  | 1.0 (ref) | 0.89 (0.45 - 1.76) | 0.79 (0.37 - 1.66) | 0.76 (0.40 - 1.44) | 0.75 (0.35 - 1.60) | 0.90 (0.76 - 1.06) | 0.343 |
| Cancer Mortality |  | 1.0 (ref) | 0.74 (0.53 - 1.02) | 0.58 (0.44 - 0.77) | 0.53 (0.38 - 0.74) | 0.44 (0.33 - 0.57) | 0.79 (0.69 - 0.90) | <0.001 |
| All-cause Mortality |  | 1.0 (ref) | 0.81 (0.68 - 0.97) | 0.72 (0.61 - 0.84) | 0.65 (0.55 - 0.77) | 0.59 (0.50 - 0.69) | 0.86 (0.81 - 0.91) | <0.001 |
| CVD Mortality | excluding serum vitamin C | 1.0 (ref) | 0.82 (0.61 - 1.10) | 0.95 (0.73 - 1.25) | 0.93 (0.70 - 1.23) | 0.95 (0.73 - 1.23) | 0.99 (0.89 - 1.10) | 0.994 |
| Heart Mortality |  | 1.0 (ref) | 0.86 (0.64 - 1.14) | 0.94 (0.70 - 1.28) | 0.98 (0.71 - 1.34) | 0.97 (0.73 - 1.30) | 1.01 (0.90 - 1.14) | 0.844 |
| Cerebral Mortality |  | 1.0 (ref) | 0.70 (0.34 - 1.44) | 0.99 (0.50 - 1.95) | 0.79 (0.43 - 1.44) | 0.84 (0.40 - 1.76) | 0.92 (0.79 - 1.08) | 0.749 |
| Cancer Mortality |  | 1.0 (ref) | 0.83 (0.57 - 1.22) | 0.77 (0.54 - 1.11) | 0.67 (0.50 - 0.90) | 0.56 (0.42 - 0.74) | 0.84 (0.73 - 0.96) | <0.001 |
| All-cause Mortality |  | 1.0 (ref) | 0.79 (0.69 - 0.90) | 0.76 (0.65 - 0.89) | 0.72 (0.63 - 0.83) | 0.62 (0.53 - 0.73) | 0.87 (0.82 - 0.93) | <0.001 |
| CVD Mortality | excluding α carotene | 1.0 (ref) | 0.83 (0.65 - 1.07) | 0.93 (0.73 - 1.2) | 0.83 (0.61 - 1.13) | 0.84 (0.65 - 1.08) | 0.98 (0.89 - 1.07) | 0.239 |
| Heart Mortality |  | 1.0 (ref) | 0.83 (0.61 - 1.13) | 0.96 (0.71 - 1.3) | 0.87 (0.64 - 1.18) | 0.85 (0.63 - 1.16) | 1.00 (0.89 - 1.12) | 0.388 |
| Cerebral Mortality |  | 1.0 (ref) | 0.82 (0.41 - 1.61) | 0.84 (0.42 - 1.72) | 0.71 (0.35 - 1.44) | 0.78 (0.39 - 1.57) | 0.91 (0.78 - 1.07) | 0.431 |
| Cancer Mortality |  | 1.0 (ref) | 0.76 (0.55 - 1.05) | 0.60 (0.44 - 0.82) | 0.60 (0.45 - 0.80) | 0.46 (0.35 - 0.60) | 0.81 (0.71 - 0.93) | <0.001 |
| All-cause Mortality |  | 1.0 (ref) | 0.78 (0.65 - 0.93) | 0.73 (0.62 - 0.85) | 0.65 (0.57 - 0.76) | 0.60 (0.51 - 0.70) | 0.87 (0.83 - 0.93) | <0.001 |
| CVD Mortality | excluding β carotene | 1.0 (ref) | 0.97 (0.76 - 1.24) | 0.90 (0.70 - 1.15) | 0.91 (0.68 - 1.21) | 0.84 (0.67 - 1.07) | 0.97 (0.87 - 1.08) | 0.186 |
| Heart Mortality |  | 1.0 (ref) | 1.01 (0.77 - 1.33) | 0.94 (0.68 - 1.29) | 0.99 (0.73 - 1.35) | 0.89 (0.67 - 1.18) | 0.99 (0.87 - 1.12) | 0.455 |
| Cerebral Mortality |  | 1.0 (ref) | 0.81 (0.40 - 1.66) | 0.78 (0.39 - 1.56) | 0.68 (0.35 - 1.31) | 0.69 (0.33 - 1.42) | 0.90 (0.75 - 1.07) | 0.223 |
| Cancer Mortality |  | 1.0 (ref) | 0.73 (0.53 - 1.00) | 0.63 (0.46 - 0.87) | 0.55 (0.39 - 0.79) | 0.46 (0.35 - 0.61) | 0.78 (0.69 - 0.87) | <0.001 |
| All-cause Mortality |  | 1.0 (ref) | 0.80 (0.68 - 0.95) | 0.72 (0.60 - 0.87) | 0.65 (0.56 - 0.76) | 0.59 (0.50 - 0.69) | 0.85 (0.80 - 0.90) | <0.001 |
| CVD Mortality | excluding Lycopene | 1.0 (ref) | 0.86 (0.61 - 1.20) | 0.94 (0.73 - 1.22) | 0.71 (0.51 - 1.00) | 0.84 (0.60 - 1.17) | 0.95 (0.86 - 1.04) | 0.139 |
| Heart Mortality |  | 1.0 (ref) | 0.92 (0.68 - 1.24) | 0.94 (0.72 - 1.22) | 0.81 (0.58 - 1.13) | 0.83 (0.59 - 1.17) | 0.96 (0.85 - 1.07) | 0.178 |
| Cerebral Mortality |  | 1.0 (ref) | 0.64 (0.28 - 1.51) | 0.96 (0.47 - 1.97) | 0.41 (0.18 - 0.94) | 0.83 (0.41 - 1.69) | 0.92 (0.79 - 1.09) | 0.513 |
| Cancer Mortality |  | 1.0 (ref) | 0.87 (0.64 - 1.19) | 0.55 (0.40 - 0.75) | 0.64 (0.48 - 0.84) | 0.47 (0.35 - 0.64) | 0.79 (0.68 - 0.92) | <0.001 |
| All-cause Mortality |  | 1.0 (ref) | 0.83 (0.70 - 0.98) | 0.77 (0.66 - 0.90) | 0.63 (0.54 - 0.73) | 0.61 (0.52 - 0.73) | 0.86 (0.81 - 0.92) | <0.001 |
| CVD Mortality | excluding Lutein/Zeaxanthin | 1.0 (ref) | 0.93 (0.66 - 1.30) | 0.91 (0.70 - 1.17) | 0.87 (0.66 - 1.15) | 0.82 (0.64 - 1.05) | 0.94 (0.86 - 1.03) | 0.107 |
| Heart Mortality |  | 1.0 (ref) | 0.95 (0.64 - 1.39) | 1.00 (0.71 - 1.40) | 0.95 (0.72 - 1.25) | 0.84 (0.62 - 1.14) | 0.96 (0.86 - 1.06) | 0.316 |
| Cerebral Mortality |  | 1.0 (ref) | 0.87 (0.46 - 1.65) | 0.61 (0.35 - 1.07) | 0.62 (0.31 - 1.25) | 0.72 (0.34 - 1.50) | 0.89 (0.74 - 1.06) | 0.261 |
| Cancer Mortality |  | 1.0 (ref) | 0.73 (0.53 - 1.01) | 0.64 (0.47 - 0.87) | 0.54 (0.39 - 0.77) | 0.50 (0.39 - 0.63) | 0.76 (0.68 - 0.84) | <0.001 |
| All-cause Mortality |  | 1.0 (ref) | 0.82 (0.69 - 0.97) | 0.72 (0.59 - 0.86) | 0.69 (0.59 - 0.79) | 0.60 (0.51 - 0.71) | 0.85 (0.80 - 0.90) | <0.001 |
| CVD Mortality | excluding β cryptoxanthin | 1.0 (ref) | 0.93 (0.69 - 1.24) | 0.91 (0.75 - 1.12) | 0.86 (0.64 - 1.16) | 0.88 (0.67 - 1.16) | 0.96 (0.87 - 1.06) | 0.306 |
| Heart Mortality |  | 1.0 (ref) | 0.91 (0.66 - 1.25) | 0.96 (0.74 - 1.26) | 0.88 (0.61 - 1.27) | 0.95 (0.70 - 1.29) | 0.99 (0.88 - 1.11) | 0.738 |
| Cerebral Mortality |  | 1.0 (ref) | 1.01 (0.50 - 2.03) | 0.75 (0.33 - 1.67) | 0.81 (0.44 - 1.49) | 0.66 (0.29 - 1.47) | 0.87 (0.73 - 1.04) | 0.147 |
| Cancer Mortality |  | 1.0 (ref) | 0.75 (0.54 - 1.05) | 0.74 (0.53 - 1.04) | 0.53 (0.37 - 0.76) | 0.47 (0.35 - 0.61) | 0.81 (0.71 - 0.92) | <0.001 |
| All-cause Mortality |  | 1.0 (ref) | 0.81 (0.68 - 0.96) | 0.76 (0.65 - 0.90) | 0.64 (0.53 - 0.76) | 0.60 (0.52 - 0.70) | 0.86 (0.81 - 0.91) | <0.001 |

Table S5 The E-value of individual biomarkers and cause-specific and all-cause mortality

| All-cause and cause-specific mortality | Biomarkers | | Hazard ratio (95% confidence interval) | | | | | |
| --- | --- | --- | --- | --- | --- | --- | --- | --- |
|  |  |  | group 1 | group 2 | group 3 | group 4 | group 5 | For each one standard deviation |
| CVD Mortality | Serum vitamin C (E-value) | |  | 2.30 | 2.12 | 2.61 | 2.45 |  |
|  |  | Model 2 | 1 | 0.68 (0.55 - 0.85) | 0.72 (0.57 - 0.92) | 0.62 (0.47 - 0.8) | 0.65 (0.5 - 0.83) | 0.93 (0.85 - 1.01) |
| Heart Mortality | Serum vitamin C (E-value) | |  | 2.61 | 2.17 | 2.90 | 2.55 |  |
|  |  | Model 2 | 1 | 0.62 (0.49 - 0.79) | 0.71 (0.54 - 0.94) | 0.57 (0.43 - 0.77) | 0.63 (0.47 - 0.85) | 0.92 (0.83 - 1.03) |
| Cancer Mortality | Serum vitamin C (E-value) | |  |  | 2.30 | 3.33 | 3.18 | 1.81 |
|  |  | Model 2 | 1 | 0.85 (0.64 - 1.12) | 0.68 (0.49 - 0.93) | 0.51 (0.35 - 0.75) | 0.53 (0.38 - 0.75) | 0.80 (0.71 - 0.91) |
|  | Serum alpha carotene (E-value) | |  | 2.21 | 2.30 | 2.50 | 3.97 | 1.92 |
|  |  | Model 2 | 1 | 0.70 (0.54 - 0.90) | 0.68 (0.48 - 0.95) | 0.64 (0.43 - 0.95) | 0.44 (0.33 - 0.60) | 0.77 (0.66 - 0.89) |
|  | Serum beta cryptoxanthin (E-value) | |  |  | 2.08 | 2.00 | 2.90 | 1.96 |
|  |  | Model 2 | 1 | 0.91 (0.72 - 1.15) | 0.73 (0.54 - 0.99) | 0.75 (0.57 - 0.99) | 0.57 (0.42 - 0.78) | 0.76 (0.65 - 0.89) |
|  | Composite biomarker score (E-value) | |  |  | 2.84 | 3.18 | 3.97 | 1.85 |
|  |  | Model 2 | 1 | 0.74 (0.53 - 1.02) | 0.58 (0.44 - 0.77) | 0.53 (0.38 - 0.74) | 0.44 (0.33 - 0.57) | 0.79 (0.69 - 0.90) |
| All-cause mortality | Serum vitamin C (E-value) | |  | 1.60 | 1.81 | 2.08 | 2.21 | 1.43 |
|  |  | Model 2 | 1 | 0.86 (0.74 - 0.99) | 0.8 (0.69 - 0.93) | 0.73 (0.62 - 0.86) | 0.70 (0.60 - 0.81) | 0.91 (0.86 - 0.96) |
|  | Total carotenoids (E-value) | |  | 1.67 | 1.88 | 2.04 | 2.35 | 1.50 |
|  |  | Model 2 | 1 | 0.84 (0.75 - 0.94) | 0.78 (0.67 - 0.90) | 0.74 (0.63 - 0.86) | 0.67 (0.58 - 0.77) | 0.89 (0.84 - 0.94) |
|  | Serum alpha carotene (E-value) | |  | 2.01 | 2.35 | 2.45 | 2.97 | 1.74 |
|  |  | Model 2 | 1 | 0.74 (0.63 - 0.87) | 0.67 (0.56 - 0.81) | 0.65 (0.54 - 0.78) | 0.56 (0.46 - 0.68) | 0.82 (0.75 - 0.89) |
|  | Serum beta carotene (E-value) | |  | 1.77 | 2.17 | 2.12 | 2.40 | 1.32 |
|  |  | Model 2 | 1 | 0.81 (0.68 - 0.97) | 0.71 (0.61 - 0.82) | 0.72 (0.62 - 0.84) | 0.66 (0.56 - 0.79) | 0.94 (0.89 - 0.99) |
|  | Serum lycopene (E-value) | |  |  | 2.08 | 1.92 | 1.96 | 1.43 |
|  |  | Model 2 | 1 | 0.89 (0.78 - 1.02) | 0.73 (0.62 - 0.87) | 0.77 (0.68 - 0.87) | 0.76 (0.65 - 0.88) | 0.91 (0.86 - 0.96) |
|  | Serum lutein/zeaxanthin (E-value) | |  |  | 1.77 | 2.08 | 2.08 |  |
|  |  | Model 2 | 1 | 0.83 (0.69 - 1.00) | 0.81 (0.7 - 0.94) | 0.73 (0.62 - 0.86) | 0.73 (0.63 - 0.85) | 0.94 (0.88 – 1.00) |
|  | Serum beta cryptoxanthin (E-value) | |  | 1.53 |  | 1.88 | 2.04 | 1.46 |
|  |  | Model 2 | 1 | 0.88 (0.78 - 0.99) | 0.86 (0.74 - 1.01) | 0.78 (0.68 - 0.91) | 0.74 (0.66 - 0.84) | 0.90 (0.86 - 0.94) |
|  | Composite biomarker score (E-value) | |  | 1.77 | 2.12 | 2.45 | 2.78 | 1.60 |
|  |  | Model 2 | 1 | 0.81 (0.68 - 0.97) | 0.72 (0.61 - 0.84) | 0.65 (0.55 - 0.77) | 0.59 (0.5 - 0.69) | 0.86 (0.81 - 0.91) |

^*^ The E-value can assess the sensitivity to unmeasured confounding.


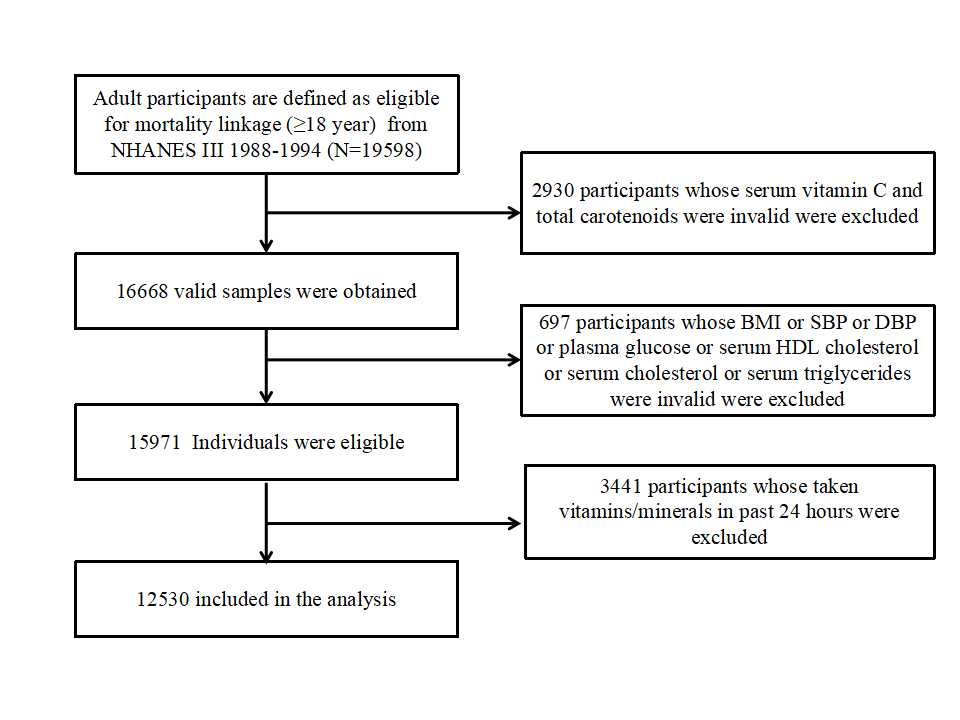


Figure S1 The flow chart of participants through the study.


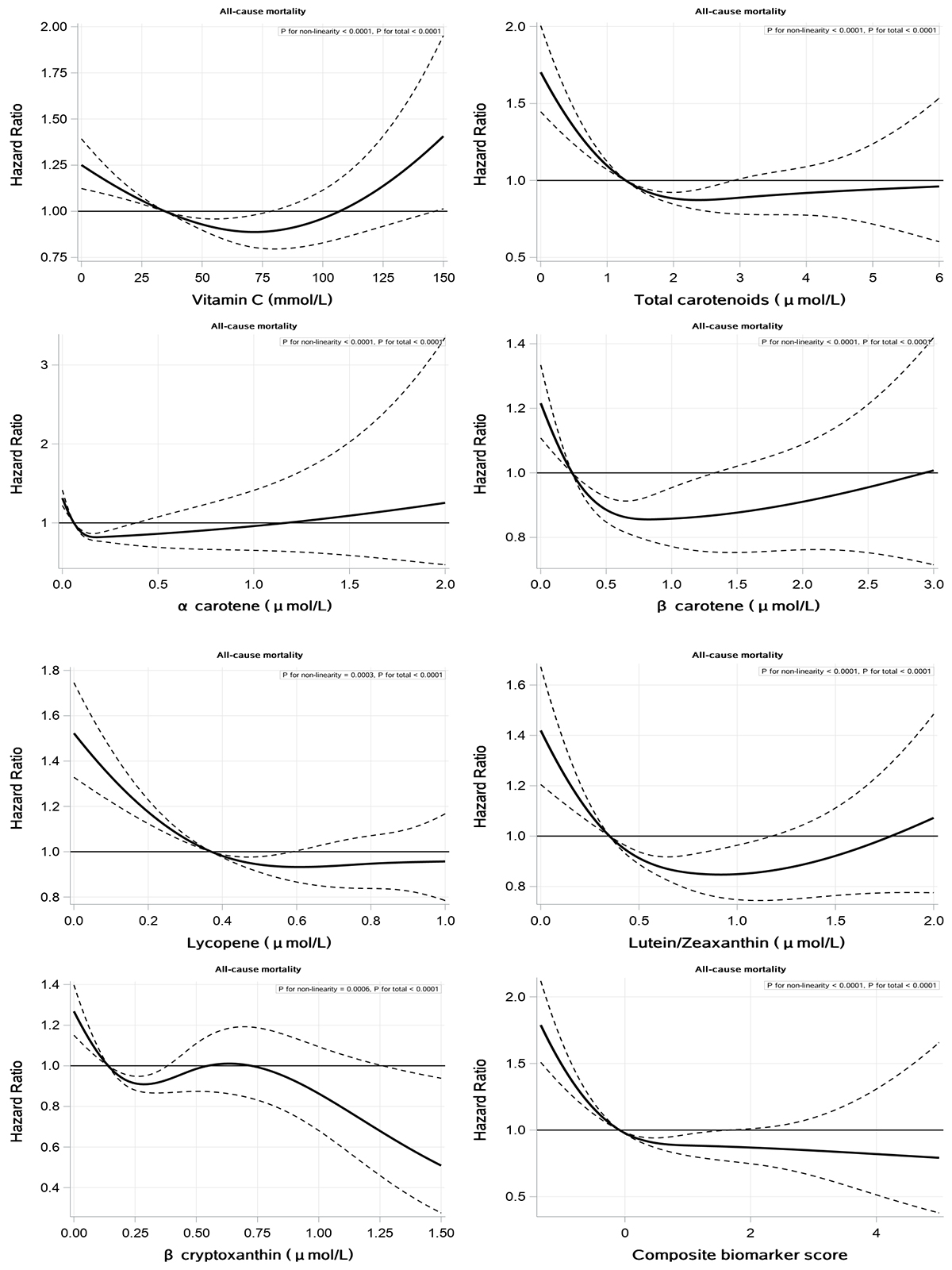


Figure S2 Associations of serum vitamin C, carotenoids, and composite biomarker score with all-cause mortality.


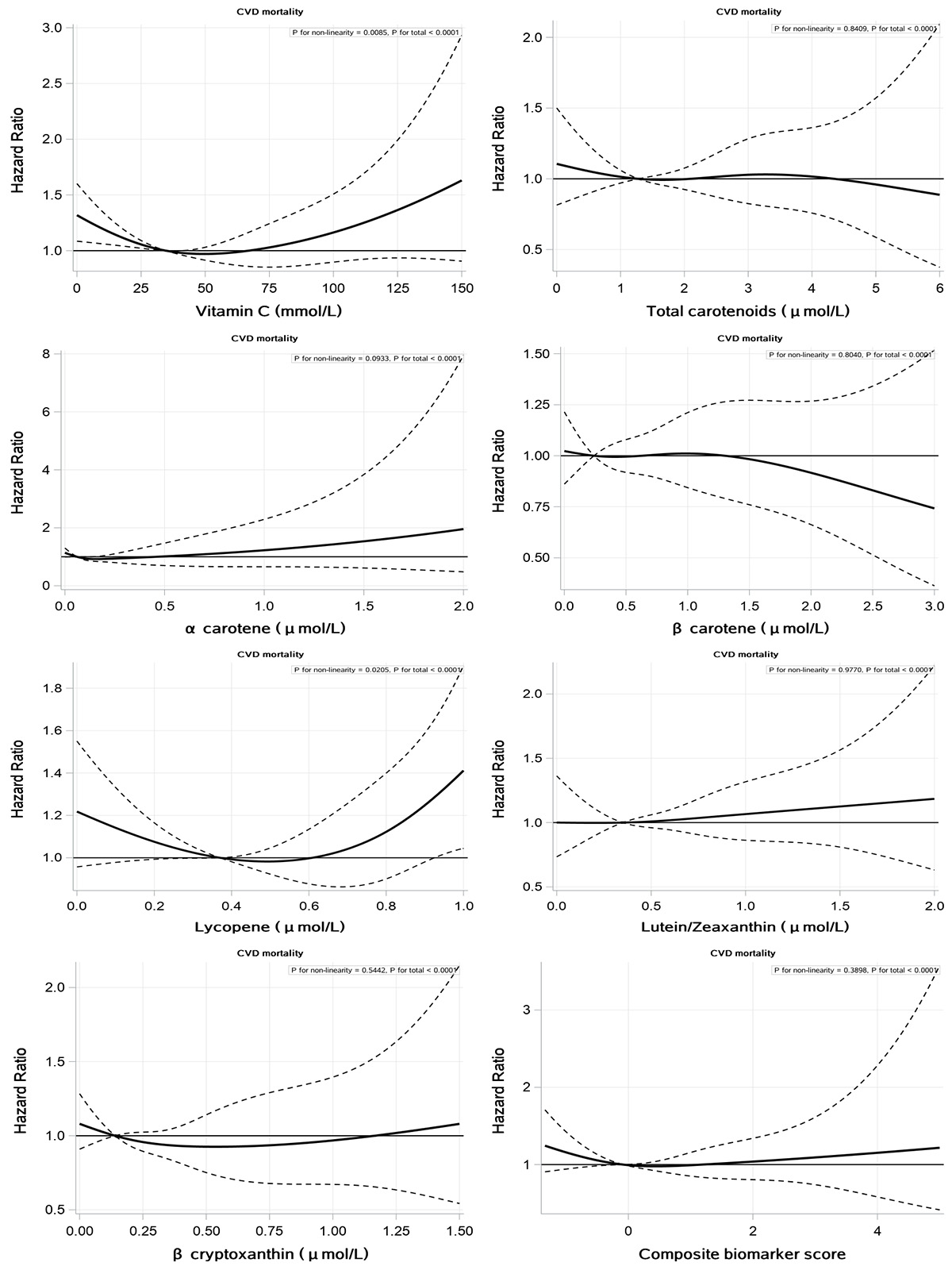


Figure S3 Associations of serum vitamin C, carotenoids, and composite biomarker score with CVD mortality.


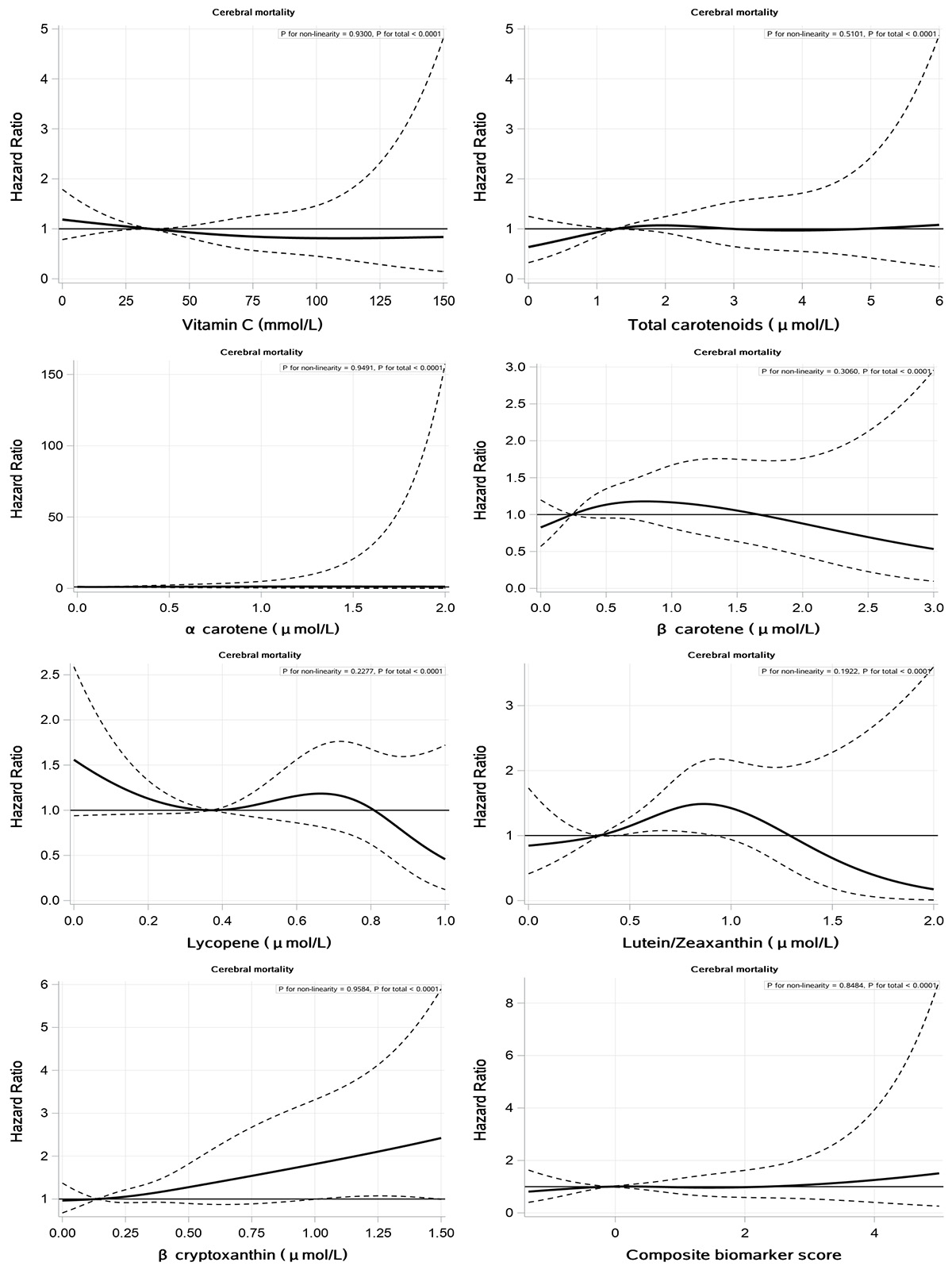


Figure S4 Associations of serum vitamin C, carotenoids, and composite biomarker score with cerebral mortality.


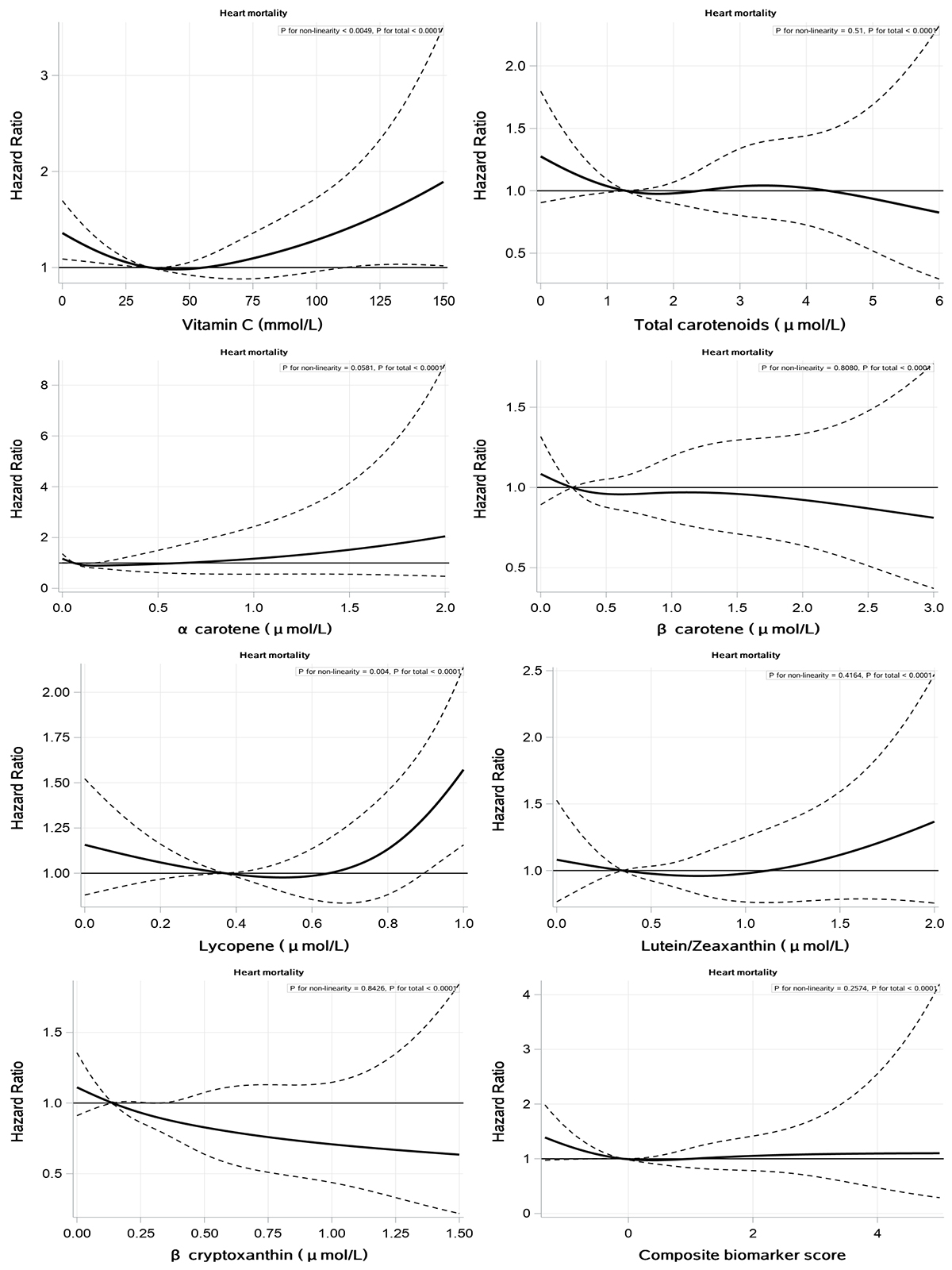


Figure S5 Associations of serum vitamin C, carotenoids, and composite biomarker score with heart mortality.
